# Raman observation of a molecular signaling pathway of apoptotic cells induced by photothermal therapy†
†Electronic supplementary information (ESI) available. See DOI: 10.1039/c9sc04389f


**DOI:** 10.1039/c9sc04389f

**Published:** 2019-10-15

**Authors:** Yingfang Xing, Zhewei Cai, Meijuan Xu, Wenzheng Ju, Xiaojun Luo, Yaojuan Hu, Xiaoyan Liu, Tuli Kang, Ping Wu, Chenxin Cai, Jun-Jie Zhu

**Affiliations:** a Jiangsu Key Laboratory of New Power Batteries , Jiangsu Collaborative Innovation Center of Biomedical Functional Materials , National and Local Joint Engineering Research Center of Biomedical Functional Materials , College of Chemistry and Materials Science , Nanjing Normal University , Nanjing 210097 , P. R. China . Email: wuping@njnu.edu.cn ; Email: cxcai@njnu.edu.cn; b State Key Laboratory of Analytical for Life Science , School of Chemistry & Chemical Engineering , Nanjing University , Nanjing 210093 , P. R. China . Email: jjzhu@nju.edu.cn; c Key Laboratory of Department of Clinical Pharmacology , Affiliated Hospital of Nanjing University of Chinese Medicine , China; d Department of Chemical and Biomolecular Engineering , Clarkson University , Potsdam , NY 13699 , USA

## Abstract

Plasmonic nanoparticle (NP)-mediated photothermal therapy (PPTT) has been explored as a minimally invasive approach to cancer therapy and has progressed from concept to the early stage of clinical trials. Better understanding of the cellular and molecular response to PPTT is crucial for improvement of therapy efficacy and advancement of clinical application. However, the molecular mechanism underlying PPTT-induced apoptosis is still unclear and under dispute. In this work, we used nuclear-targeting Au nanostars (Au NSs) as both a photothermal agent to specifically induce apoptosis in cancer cells and as a surface enhanced Raman spectroscopy (SERS) probe to monitor the time-dependent SERS spectra of MCF-7 cells which are undergoing apoptosis. Through SERS spectra and their synchronous and asynchronous SERS correlation maps, the occurrence and dynamics of a cascade of molecular events have been investigated, and a molecular signaling pathway of PPTT-induced apoptosis, including release of cytochrome c, protein degradation, and DNA fragmentation, was revealed, which was also demonstrated by metabolomics, agarose gel electrophoresis, and western blot analysis, respectively. These results indicated that PPTT-induced apoptosis undergoes an intrinsic mitochondria-mediated apoptosis pathway. Combined with western blot results, this intrinsic mitochondria-mediated apoptosis pathway was further demonstrated to be initiated by a BH3-only protein, BID. This work is beneficial for not only improving the fundamental understanding of the molecular mechanism of apoptosis induced by PPTT but also for guiding the modulation of PPTT to drive forward its clinical application.

## Abstract 2

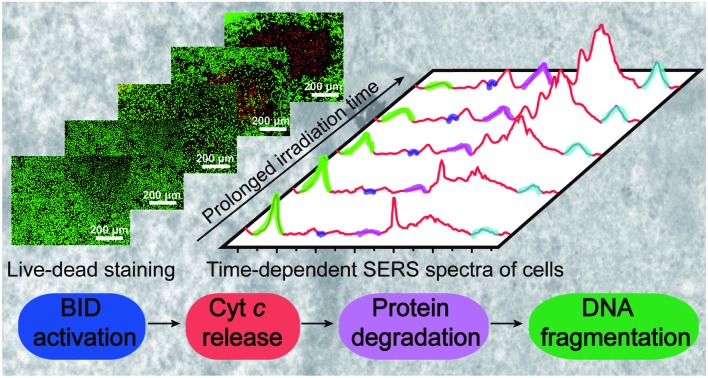
A molecular signaling pathway of apoptosis induced by photothermal therapy was revealed by surface-enhanced Raman spectroscopy.

## Introduction

PPTT has been explored as a minimally invasive approach to cancer therapy. This form of cancer therapy is achieved by killing cancer cells *via* localized hyperthermia converted from light absorption with the use of plasmonic NPs that are previously loaded into the cancerous cells. Considerable efforts have been focused on the design and synthesis of plasmonic NPs as PPTT agents over the past few decades. A variety of photothermal conversion agents have been reported, including organic compounds (*e.g.*, indocyanine green^1^ and polyaniline^2^), Au NPs (*e.g.*, Au nanospheres,^3^ Au nanorods,^4^ Au nanocages,^5^ and branched Au nanoparticles^6^), carbon-based materials (*e.g.*, graphene oxide,^7^ carbon nanotubes,^8^ and graphydine^9^), and some metal sulfide NPs.^10^,^11^ Of these plasmonic NPs, Au NPs show the most favorable performance in killing cancer cells and ablation of tumors owing to their profound and controllable localized surface plasmon resonance (LSPR) in the NIR region and fairly high biological inertness, and also no major ion dissolution occurs under biological conditions.^12^ The use of AuNPs has moved PPTT to the early stage of clinical trials.^13^,^14^

To further drive forward the clinical application of PPTT, it is crucial to unravel the cellular response and molecular response to PPTT because they are considerably helpful in pursuing maximized therapy efficacy and minimized undesirable side effects. Some recent studies have carried out investigations of the cellular response to PPTT with the use of Au NPs.^15^–^20^ Although apoptotic and necrotic pathways were both reported, it is commonly accepted that apoptosis is a more favorable pathway than necrosis, and apoptosis can be selectively induced under suitable PPTT conditions, such as the use of Au NPs with high photothermal conversion efficiency, active targeting, and moderate light dosage (*e.g.* low irradiation power density and short irradiation duration).^18^–^20^

However, the molecular response, especially the molecular mechanism of PPTT-induced apoptosis, still remains largely unknown and under dispute. El-sayed and coworkers previously conducted PPTT in three different epithelial cancer cell lines including HSC (oral), MCF-7 (breast) and Huh7.5 (liver), and observed *via* immunoblotting that their response to PPTT is correlated with a heat-shock protein (HSP70), an upstream inhibitor of apoptosis which inhibits by preventing cytochrome c/dATP-mediated caspase activation.^21^ The lower the initial HSP70 level, the higher the population of apoptotic cells induced by PPTT. Recently, with the use of SERS measurements combined with metabolomics and proteomics experiments, this group observed an increase in the level of phenylalanine and its derivatives in HSC cells after PPTT, and proved the disorder in phenylalanine metabolism within mitochondria-mediated apoptosis through Rho/ROCK-associated kinase and the Fas/Fas ligand death receptor pathway.^22^ del Pino and colleagues by using biological reporters (Annexin V and 7-aminoactinomycin D) coupled with flow cytometry assays also observed mitochondria-mediated apoptosis in murine embryonic fibroblast (MEF) cells treated with PPTT.^23^ However, they found that the mitochondrial pathway of apoptosis is mediated by the nuclear-encoded proteins Bak and Bax through the activation of BID protein. These results are conflicting. An acknowledged molecular mechanism of PPTT-induced apoptosis is still challenging to discover. Moreover, although the typical molecular events and their kinetics in PPTT-induced apoptosis are significantly important to regulate the process of apoptosis, they have not yet been studied in detail.

Herein, we used the SERS technique to *in situ* collect the time-dependent SERS spectra of cells which were undergoing PPTT-induced apoptosis, through which we can observe the molecular events and obtain their dynamic information in real time, and further unravel the molecular signaling pathway of PPTT-induced apoptosis. Nuclear-targeting Au nanostars (Au NSs) were used as both PPTT agents and SERS probes because Au NSs have been demonstrated to possess a considerably high photothermal conversion efficiency (56%) for converting 808 nm near-infrared (NIR) light to heat in our previous work,^24^ and also can produce a tremendous enhancement in SERS activity.^25^,^26^ We constructed nuclear-targeting Au NSs and loaded them into living cells, where they can selectively localize within the perinuclear region and thereafter considerably enhance Raman signals from the nuclei in the physiological environment. We followed the time-dependent SERS spectra of cells undergoing PPTT, through which the molecular events responding to PPTT can be observed. We further investigated the dynamics of these molecular events by using a synchronous and asynchronous SERS correlation analysis. An intrinsic mitochondria-mediated apoptosis pathway, where a cascade of molecular events, including the release of cytochrome c, protein degradation, and DNA fragmentation occurs, was thus elucidated. Together with western blot analysis, this mitochondria-mediated apoptosis pathway was indicated to be initiated by the BH3-only protein BID. This result is beneficial for not only improving the fundamental understanding of the intracellular signaling cascades activated by PPTT but also for guiding the modulation of PPTT to advance its clinical application.

## Results and discussion

### Functionalization and cellular uptake of nuclear-targeting Au NSs

**Fig. 1:**
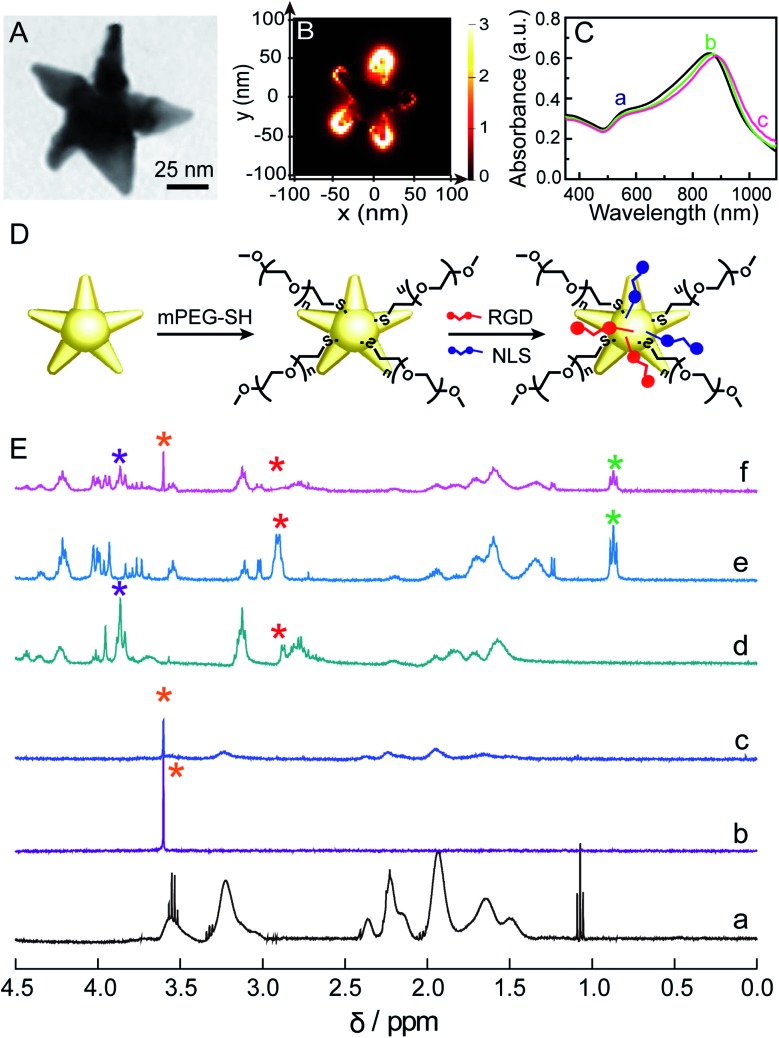
(A) TEM image of the Au NSs. (B) FDTD-simulated electric field distribution of the Au NSs at 785 nm. (C) UV-vis-NIR spectra of the Au NSs (a), mPEG-Au NSs (b), and mPEG/RGD/NLS-Au NSs (c). (D) Schematic illustration of the functionalization of the mPEG/RGD/NLS-Au NSs. (E) 1H NMR spectra of the 0.0–4.5 ppm region of the PVP-Au NSs (a), mPEG (b), mPEG-Au NSs (c), RGD (d), NLS (e) and mPEG/RGD/NLS-Au NSs (f).

We used a seed-mediated growth method to synthesize Au NSs.^24^ The as-synthesized Au NSs show a central core and 5 protruding arms with sharp tips (Fig. 1A and S1 in the ESI†). The mean core diameter is 20 ± 2 nm, and the average length of the arms is 36 ± 3 nm. This particle size is favorable for endocytosis.^27^ The sharp tips can work as antennas to enhance the SERS activity of Au NSs, as confirmed by Finite-Difference Time-Domain (FDTD) simulation results, in which an intensive electromagnetic field was observed at the tip (Fig. 1B), as well as the amplitude of enhancement of the field achieved was ∼3.0 × 10^3^ at 785 nm (Fig. S2†). Given that a 785 nm near-infrared laser will be used herein as an excitation source to prevent the auto-fluorescence emitted from living cells, such a significant electromagnetic enhancement at 785 nm will greatly enhance the cellular Raman signals. We theoretically evaluated the SERS enhancement factor (EF) of the Au NSs to be ∼1.0 × 10^7^ as the EF is proportional to the fourth power of the ratio of the local electric field at 785 nm to the incident electric field ((*E*_max_/*E*_0_)^4^). To quantify the additional contributions of all inelastic scattered photons interacting with the plasmonic nanoparticles, a quality factor (*Q*_F_) was adapted from Blaber *et al.*^28^ The *Q*_F_ was calculated to be ∼1.0 × 10^8^ (please refer to the calculation details in the ESI†). Altogether, these results strongly suggest that the Au NSs as SERS probes have promising potential in sensitively sensing the molecular response to PPTT within the cells.

Before using Au NSs as both a photothermal conversion agent and SERS probe for *in vitro* PPTT, we engineered Au NSs *via* surface modification to improve their biocompatibility and nuclear-targeted cellular uptake. Towards this goal, we first coated the Au NSs with methoxypolyethylene glycol thiol (mPEG-SH) to ensure stability in a biological environment. Moreover, the presence of mPEG can prevent unspecific adsorption of proteins present in physiological media.^29^ Then, we modified mPEG-coated Au NSs with a cell-penetrating peptide, RGD (binds to integrin expressed on cell membranes to enhance cell/biomaterial interaction^30^), and nuclear localization signal (NLS) peptides (interact with importin and translocate near the nucleus^31^) to facilitate endocytosis and nuclear targeting (Fig. 1D). This is because plasmonic nanomaterials located in the perinuclear region can not only lead to maximum cell destruction through apoptosis^18^ but can also better monitor the biochemical events associated with genetic substances in the nucleus. The successful coating of the Au NSs with mPEG, RGD, and NLS peptides was evidenced by a continuous redshift of the main LSPR peak from 857 nm for the Au NSs to 873 nm for the mPEG/Au NSs and finally to 883 nm for the mPEG/RGD/NLS-Au NSs (Fig. 1C). The stepwise functionalization was also confirmed from the zeta potential values, which were –21.7 mV for the Au NSs, –19.7 mV for the mPEG/Au NSs, and –11 mV for the mPEG/RGD/NLS-Au NSs after mPEG and peptide modification. Furthermore, the functionalization was investigated by NMR spectroscopy. As shown in Fig. 1E, we compared the ^1^H NMR spectra of PVP-Au NSs (curve a) and mPEG (curve b) with those of mPEG-Au NSs (curve c), and obviously observed a new peak present at 3.60 ppm, corresponding to –CH_2_ groups in mPEG,^32^ as well as a significant diminishment in signals for PVP, indicating the successful substitution of PVP with mPEG. After modification of the surface of mPEG-Au NSs with RGD and NLS, changes can also be seen in the ^1^H NMR spectrum of the mPEG/RGD/NLS-Au NSs (curve f) relative to free RGD (curve d) and NLS (curve e). The signals of peptides (3.92–3.77 and 0.94–0.80 ppm) can be easily recognized in the spectrum of the mPEG/RGD/NLS-Au NSs. Moreover, the peak for –CH_2_ groups in mPEG was also observed at 3.60 ppm. It is worth noting that the peak assigned to β-CH_2_ groups of cysteine in RGD and NLS (2.90 ppm) vanished after assembly on mPEG-Au NSs. This is commonly observed for signals that are due to hydrogen atoms present very close to the gold surface,^33^ implying that the peptides were assembled. Moreover, the peptides assembled on mPEG-Au NSs slightly change the location and shape of chemical shifts of residues compared to the free peptides, suggesting the successful coating of RGD/NLS on mPEG-Au NSs. This should be due to the interaction between mPEG chains and RGD/NLS. Previous work had demonstrated that long PEG chains are likely to fold themselves to interact with some amino acid residues in protein.^34^ As a result, from the UV-vis and NMR spectra, along with the alterations in zeta potential values, the successful functionalization of the Au NSs with mPEG and peptides can be confirmed.

Next, we investigated the cellular internalization of the mPEG/RGD/NLS-Au NSs. First, the colloidal stability of the modified Au NSs in culture medium was investigated. Stable dispersions of the mPEG/RGD/NLS-Au NSs can be produced in culture medium, as evidenced by the invariable UV-vis spectra of the mPEG/RGD/NLS-Au NSs in culture medium over 1 week (Fig. S3†). This should be contributed from the attachment of mPEG to the particle surface, which has been proven to be particularly effective for improving nanoparticle stabilization in a nonaggregated state in the presence of high concentration of salt and biomolecules.^35^ The zeta potential values of the mPEG/RGD/NLS-Au NSs remained negative in culture medium (–7.4 mV), showing that the mPEG/RGD/NLS-Au NSs have negative surface charges which can facilitate their slow uptake by cells *via* the endocytosis route and low toxicity.^36^,^37^ As indicated by the 3-(4,5-dimethylthiazol-2-yl)-2,5-diphenyltetrazolium bromide (MTT) assay results, there was no evident cytotoxicity of the mPEG/RGD/NLS-Au NSs at a concentration of 100 μg mL^–1^ (Fig. S4†). Therefore, 100 μg mL^–1^ was chosen as the treatment concentration of the mPEG/RGD/NLS-Au NSs in the following *in vitro* experiments.

The internalization was first shown by the UV-vis-NIR spectra of culture medium with the mPEG/RGD/NLS-Au NSs before and after incubation with cells. As indicated in Fig. S5,† after incubation of the cells with the nanoparticles, the intensity of the LSPR peaks of the Au NSs sharply decreases which reflects that a portion of Au NSs are being uptaken by the cells. We further quantified the cellular uptake amount to be ∼6.5 fg Au per cell by inductively coupled plasma (ICP) measurements.

**Fig. 2:**
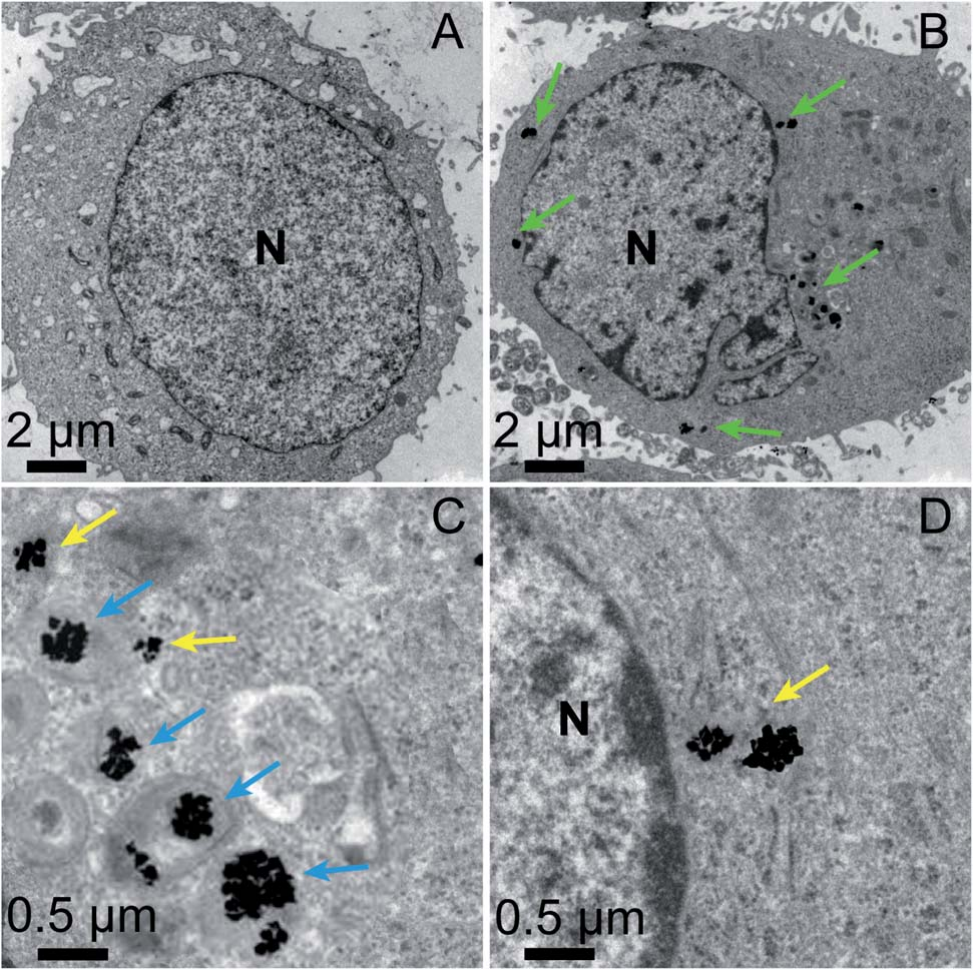
TEM images of an ultrathin slice of MCF-7 cells without (A) and with (B) incubation in the presence of the mPEG/RGD/NLS-Au NSs (100 μg mL^–1^) for 24 h. (C and D) TEM images of the selected areas in panel B at a high magnification. N: nucleus. mPEG/RGD/NLS-Au NSs can be observed in the perinuclear region (green arrows), in lysosomes (blue arrows), outside the lysosomes and close to the nucleus (yellow arrows).

The location of the mPEG/RGD/NLS-Au NSs inside the cells was confirmed by confocal laser scanning microscopy (CLSM) images (Fig. S6†). We labeled the mPEG/RGD/NLS-Au NSs with Cy5 (emits red signals) and stained the cells with a nucleus marker, Hoechst 33342 (emits blue signals). In the overlaid image, we can see that the red signals surround the blue one, indicative of the presence of the Au NSs in the perinuclear region. And we can observe this distribution from a z-stack image. The exact localization of the Au NSs was further revealed by TEM images. MCF-7 cells incubated without and with the mPEG/RGD/NLS-Au NSs were fixed, sectioned, and imaged (Fig. 2A–D). Fig. 2B shows that the mPEG/RGD/NLS-Au NSs are distributed in the cytosol around the nucleus. The high-magnification TEM images further show that some of the Au NSs are enclosed in the lysosomes (darkest endosomes) (Fig. 2C), and some others are close to the nucleus (Fig. 2D), implying that these Au NSs had escaped from the endosomes and moved to the perinuclear region under the drive of the nuclear-targeting peptide. All the results confirm the nuclear-targeted cellular uptake.

### Molecular events in PPTT-induced apoptosis

The mPEG/RGD/NLS-Au NSs were then used as heaters and SERS probes in *in vitro* PPTT experiments. MCF-7 cells were cultured with 100 μg mL^–1^ mPEG/RGD/NLS-Au NSs for 24 h, and then exposed to an 808 nm laser (0–1 W cm^–2^) for 0–15 min. We found that temperature increases in cells showed laser power density-dependency and irradiation time-dependency (Fig. S7†). Typically, greater increases were observed for higher laser power densities and longer irradiation times. When the cells were exposed to the 808 nm laser with a power density of 0.5 W cm^–2^ for 15 min, the temperature in cells increased to 45 °C. Given that the threshold temperature for inducing cellular damage is 41–47 °C and there is a need to minimize unnecessary heating of normal tissues (<45 °C),^38^ combined with the low skin tolerance threshold, an irradiation power density of 0.5 W cm^–2^ and irradiation time of 15 min were specifically chosen in the following PPTT experiments.

The therapeutic effect on MCF-7 cells was first investigated with the live–dead staining results, which show that a satisfactory therapeutic efficacy was achieved (Fig. S8†). The MTT analysis reveals that the death ratio of PPTT-treated cells finally reached 98.0% (Fig. S9†). To determine whether apoptosis or necrosis was occurring, we investigated the morphology, trypan blue staining, lactate dehydrogenase (LDH) release, and the caspase 3 activity of the PPTT-treated cells (Fig. S10†). The differential interference contrast (DIC) images clearly display the shrinkage of the cells, formation of membrane blebbing and apoptotic bodies (Fig. S10A†), which are typical morphological features of apoptotic cells. The cells also show trypan blue stain-resistance (Fig. S10B†), indicative of their membrane integrity, another important feature of apoptotic cells. LDH release, as an indicator of membrane damage, remained constant following high-energy irradiation (Fig. S10C†), again indicating the integrity of the membrane. In contrast, the caspase 3 activity showed an increased level during the irradiation (Fig. S10D†). These results accumulatively demonstrate that apoptosis was induced (please refer to the ESI† for details). The apoptosis was also confirmed by a flow cytometry measurement. PPTT-treated cells were stained with propidium iodide (PI) and fluorescein isothiocyanate (FITC)-conjugated Annexin V (Annexin V-FITC). Late apoptotic cells are Annexin V/PI-double-positive. As can be seen in Fig. S11 and S12,† the number of apoptotic cells increases with prolonging the irradiation time and finally dominates for the population of dead cells.

Meanwhile, we monitored the SERS spectra *in vitro* as a function of irradiation time during apoptosis by using mPEG/RGD/NLS-Au NSs loaded within cells as SERS probes. First, we compared the SERS spectra of mPEG/RGD/NLS-Au NSs before and after loading into MCF-7 cells. As indicated by the different profiles of the SERS spectra in Fig. S13,† the surface modification of Au NSs by mPEG and peptides shows no effect on the SERS signals from MCF-7 cells. Moreover, we evaluated an SERS enhancement factor (*E*_F_) of ∼1.1 × 10^6^ for the mPEG/RGD/NLS-Au NSs by using Rh 6G as a model analyte (Fig. S14†), implying that the Au NSs also have high SERS activity after surface modification. The result benefits the use of the mPEG/RGD/NLS-Au NSs as probes *in vitro*. Furthermore, we compared the SERS activity of the mPEG/RGD/NLS-Au NSs before and after exposure to an 808 nm laser (0.5 W cm^–2^) for 15 min. The heated mPEG/RGD/NLS-Au NSs produced the same SERS signal as they did before irradiation (Fig. S15†), indicating that the Au NSs can maintain their shape under such a moderate irradiation. This should be because the PEG and peptides attached on the surface of the Au NSs can work as a protective amorphous carbon layer to prevent deformation of Au NSs upon heating,^39^ as well as because a low-energy irradiation strategy (0.5 W cm^–2^) was used in our study with a moderate temperature elevation (45 °C), under which no reshaping occurred. This result shows that the mPEG/RGD/NLS-Au NSs can enable a reproducible SERS signal during the PPTT process when using our irradiation conditions.

**Fig. 3:**
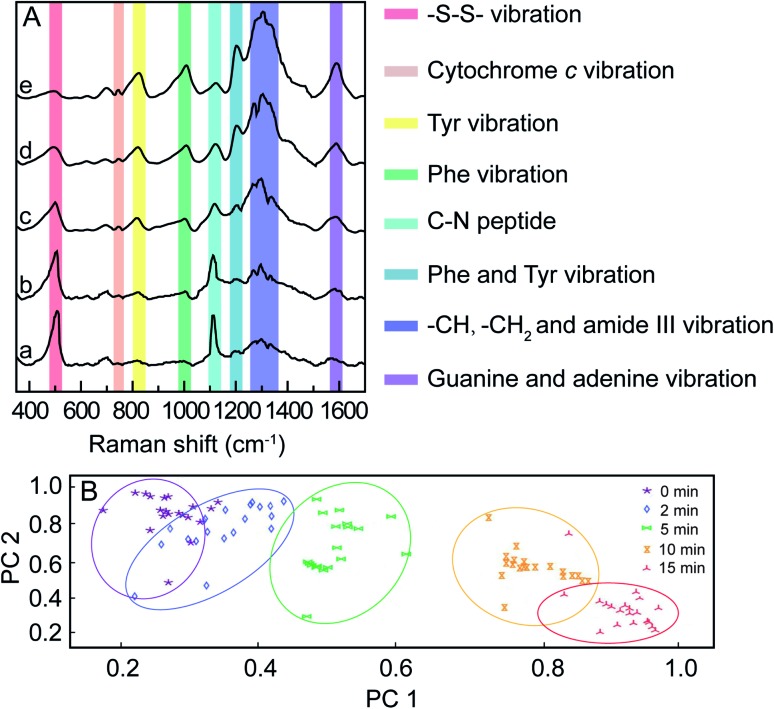
(A) Normalized SERS spectra of MCF-7 cells incubated with the mPEG/RGD/NLS-Au NSs under an 808 nm laser (0.5 W cm^–2^) for 0 (a), 2 (b), 5 (c), 10 (d), and 15 min (e). (B) PCA analysis of 30 SERS spectra of MCF-7 cells exposed to an 808 nm laser (0.5 W cm^–2^) at different times during the PPTT process. The excitation used in SERS experiments was 785 nm.

For *in vitro* SERS measurement, cells seeded on a silicon slice were incubated with 100 μg mL^–1^ mPEG/RGD/NLS-Au NSs for 24 h and then irradiated with an 808 nm NIR laser (0.5 W cm^–2^). SERS spectra were collected at different irradiation times using 785 nm laser excitation. Fig. 3A shows the normalized SERS spectra of the MCF-7 cells during the PPTT process, where some characterized Raman bands of cellular components are discerned, such as the band at 500 cm^–1^ assigned to the –S–S– stretching vibration,^40^–^42^ 750 cm^–1^ assigned to the pyrrole breathing mode *ν*_15_ in cytochrome c,^43^ 1120 cm^–1^ ascribed to the stretching vibration of the C–N peptide bond in proteins,^44^ 825 cm^–1^ ascribed to out-of-plane ring breathing corresponding to tyrosine (Tyr),^45^ 1000 cm^–1^ attributed to benzene ring breathing of phenylalanine (Phe),^46^–^48^ 1207 cm^–1^ attributed to C_6_H_5_–C stretching of Phe and Tyr,^49^,^50^ 1250–1350 cm^–1^ attributed to stretching vibrations of –CH, –CH_3_, and amide III,^51^ and 1586 cm^–1^ assigned to the N–H bending vibration of guanine or adenine residues within DNA.^52^ We observed that dying and apoptotic cells have notable spectroscopic changes compared to living cells. The intensities at 500 and 1120 cm^–1^ decrease, while the intensities at 750, 825, 1000, 1207, 1250–1350, and 1586 cm^–1^ increase with the prolonging of irradiation time.

These spectroscopic characteristic changes can be highlighted by principal component analysis (PCA). As depicted in Fig. 3B, the corresponding plots for PC1 and PC2 reveal a clear segregation between MCF-7 cells for various irradiation times. When plotting the score values of the SERS spectra of MCF-7 cells before and after 15 min of NIR irradiation, the PC1 score value was 59%, indicating that 59% of the total spectral variance significantly differs. Using the PC2 scores, irradiated cells were also clearly separable from cells before irradiation, where PC2 accounted for 31% of the total spectral variance. This result demonstrates that the changes in the cellular spectroscopic characteristics experienced during the apoptotic process can be distinctly discriminated.

**Fig. 4:**
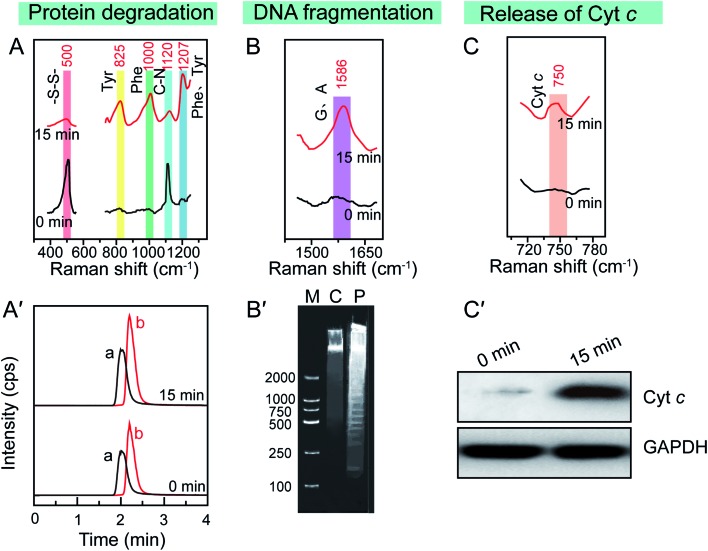
Typical apoptotic molecular events in PPTT-induced apoptosis. *In situ* reference Raman bands of (A) protein degradation, (B) DNA fragmentation, and (C) release of cytochrome c in the SERS spectra of MCF-7 cells before (up) and after (bottom) PPTT. (A′) LC-MS analysis of Phe and Tyr extracted from MCF-7 cells before (up) and after (bottom) PPTT. (B′) Agarose gel electrophoresis of DNA extracted from MCF-7 cells before and after PPTT. (C′) Agarose gel electrophoresis of DNA fragments. Lane M: DNA ladder mark in the range of 100–2000 bp, lane C: control (DNA fragments extracted from MCF-7 cells before PPTT), lane P: DNA fragments extracted from MCF-7 cells after PPTT.

Since the enhanced cellular Raman signals are only observed in the surrounding of the Au NSs, the time-dependent SERS spectra of cells during the PPTT thus can provide information on the molecular events that occur around the Au NSs when heat is generated in real time. We thus carefully studied the molecular events occurring during the PPTT-induced apoptosis. As shown in Fig. 4A, the decreased intensity at 500 cm^–1^ is well known to be attributed to the breakage of disulfide bonds,^40^–^42^ indicating the loss of the tertiary structure of a protein during the PPTT process due to the breakage of disulfide bonds which are significantly important to keep the native folding structures of proteins. This unfolding of protein can also be evidenced by the intensity changes within 1250–1350 cm^–1^ corresponding to alterations in amide.^51^ The decreased intensity at 1120 cm^–1^ refers to the breakage of peptide bonds,^44^ indicating the hydrolysis of peptide bonds during protein degradation. Meanwhile, the parallel intensity increases of bands at 825, 1000, and 1207 cm^–1^, reference bands for Phe and Tyr, are indicators of the increased amount of intracellular amino acids.^45^–^50^ As indicated by these changes of Raman bands, we can discern the molecular events including protein unfolding, peptide hydrolysis, and release of amino acids, which are together indicative of protein degradation, a typical molecular event of apoptosis, in which executioner caspases 3 are activated which then kill the cells by indiscriminately degrading proteins.^53^

To confirm the protein degradation, we carried out metabolomics experiments to examine the released amino acids, which are the final products of protein degradation. The metabolites of PPTT-treated MCF-7 cells at different irradiation time intervals were extracted. LC-MS analysis was used to measure the levels of Phe and Try. An uptrend in the levels of Phe and Tyr was found to be associated with the prolonged irradiation time (Fig. 4A′ and S16†), providing further evidence of the occurrence of protein degradation.

Aside from protein degradation, we also revealed many other molecular events through time-dependent SERS spectra of the PPTT-treated MCF-7 cells. For example, the SERS spectra show an intensity increase at 1586 cm^–1^ after PPTT (Fig. 4B). Since this intensity increase is attributed to more exposure of adenine and guanine,^53^ it signals the DNA fragmentation caused by PPTT. Agarose gel-electrophoresis further provided solid evidence, in which DNA samples extracted from MCF-7 cells after 15 min irradiation display a ladder (lane P in Fig. 4B′); in contrast, those extracted from MCF-7 cells before irradiation show a dispersive band (lane C in Fig. 4B′). This comparison verified DNA fragmentation in MCF-7 cells after PPTT.

The Raman band at 750 cm^–1^ is assigned to cytochrome c.^43^ Upon PPTT, the intensity at 750 cm^–1^ increased (Fig. 4C), indicating more release of cytochrome c during the PPTT process, an important characteristic feature of mitochondria-mediated apoptosis. Similar to the protein degradation and DNA fragmentation profiles, we carried out *in vitro* experiments to confirm it. We extracted cytochrome c from the cytoplasm of MCF-7 cells before and after PPTT, and performed western blot analysis to compare their expression levels. As shown in Fig. 4C′, a significantly elevated level of cytochrome c in the cytoplasm appears after PPTT, confirming the PPTT-induced release of cytochrome c and validating the SERS findings again.

Altogether, these observed characteristics demonstrate that a chain of molecular events, including protein degradation, DNA fragmentation, and cytochrome c release, occur during the PPTT process, which are characteristic molecular responses to apoptosis.^54^–^56^ The same SERS experiments were repeated using A549 and HeLa cells. The same trends of Raman peak intensity changes at 500, 750, 825, 1000, 1120, 1207, 1250–1350, and 1586 cm^–1^ were obtained (Fig. S17 and S18†), indicating that the molecular events in PPTT-induced apoptosis are consistent across different cell lines.

### Dynamics of molecular events in PPTT-induced apoptosis

**Fig. 5:**
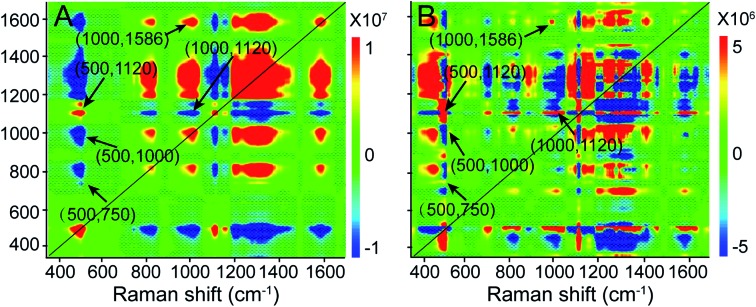
(A) 2D synchronous and (B) asynchronous Raman correlation maps for the region 350–1700 cm^–1^. The color bar shows the different intensities of the 2D correlation peaks.

Additionally, we performed a 2D correlation analysis to study the dynamics of the molecular events that occur during PPTT-induced apoptosis. Fig. 5A and B show the synchronous and asynchronous 2D correlation maps in the region of 350–1700 cm^–1^, where the bands corresponding to –S–S– (500 cm^–1^), peptide bonds (1120 cm^–1^), Phe (1000 cm^–1^), adenine and purine (1586 cm^–1^), and cytochrome c (750 cm^–1^) are highlighted because they are reference bands for protein degradation, DNA fragmentation, and cytochrome c release. We observed that the cross peaks at (500, 1120) and (1000, 1586) cm^–1^ are positive in both the synchronous and asynchronous spectra; the cross peak at (500, 1000) cm^–1^ is negative in both the synchronous and asynchronous spectra, and the cross peaks at (500, 750) and (1000, 1120) cm^–1^ are negative in the synchronous spectrum but positive in the asynchronous spectrum (the details are summarized in Table 1).

**Table 1:** Order of band changes during the PPTT process

Synchronous at *Φ* (*ν*_1_, *ν*_2_)	Asynchronous at *ψ* (*ν*_1_, *ν*_2_)	*ν*_1_ ^*a*^
(500, 750) cm^–1^ < 0	(500, 750) cm^–1^ > 0	Lag
(500, 1000) cm^–1^ < 0	(500, 1000) cm^–1^ < 0	Lead
(500, 1120) cm^–1^ > 0	(500, 1120) cm^–1^ > 0	Lead
(1000, 1120) cm^–1^ < 0	(1000, 1120) cm^–1^ > 0	Lag
(1000, 1586) cm^–1^ > 0	(1000, 1586) cm^–1^ > 0	Lead

^*a*^The term lead means that the intensity change of a band at *ν*_1_ occurs before *ν*_2_. The term lag means that the intensity change of a band at *ν*_1_ occurs after *ν*_2_.

According to Noda's rules,^57^ we conclude that the order of band changes is as follows: the change of the 750 cm^–1^ band first begins to show up, followed by changes in the bands at 500, 1120, and 1000 cm^–1^, and lastly, the 1586 cm^–1^ band changes. Among them, the changes in the 500, 1120, and 1000 cm^–1^ bands are related to protein degradation. Their occurrence sequence is as follows: 500, 1120, and 1000 cm^–1^, indicating that in protein degradation breakage of the disulfate bonds first occurs, which leads to unfolding of the protein, and then hydrolysis of the unfolded proteins takes place, and finally hydrolysates and amino acids are released. Following the 1000 cm^–1^ band, the intensity vibration at the 1586 cm^–1^ band changes, which is the reference band for DNA fragmentation, indicating that DNA fragmentation is a downstream event of protein degradation. This sequence agrees well with a previous publication,^58^ in which the 500 cm^–1^ band was designated as the “spectroscopic death initiation” band and the 1586 cm^–1^ band was called the “SERS death” band. In this study, we highlighted the Raman band corresponding to cytochrome c (750 cm^–1^) in particular because its release is a significant feature of mitochondria-mediated apoptosis. We found that the change at 750 cm^–1^ was the first to occur, even prior to that at 500 cm^–1^, indicating that the release of cytochrome c is upstream of protein degradation, which is in good agreement with the result obtained by the western blot method.^59^ Using information provided by the 2D correlation maps, we thus conclude that a cascade of molecular events occurs, and elucidate the molecular signaling pathway in PPTT-induced apoptosis: release of cytochrome c, degradation of protein (including unfolding of protein, hydrolysis of peptide bonds, and release of amino acids), and fragmentation of DNA.

**Fig. 6:**
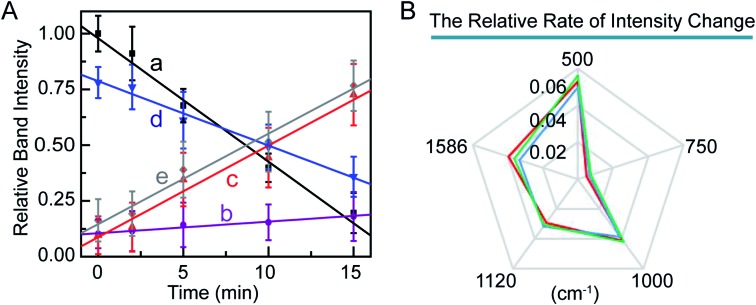
(A) Relative Raman band intensities at 500 (a), 750 (b), 1000 (c), 1120 (d), and 1586 cm^–1^ (e) in the SERS spectra of MCF-7 cells as a function of irradiation time. (B) Map of the relative rate of intensity changes of bands at 500, 750, 1000, 1120, and 1586 cm^–1^ in the SERS spectra of MCF-7 cells (red curve), A549 (green curve), and HeLa (blue curve).

Furthermore, we studied the kinetics of these molecular events in the PPTT-induced apoptosis. We monitored the changes in band intensities at 500, 750, 1000, 1120, and 1586 cm^–1^ as a function of NIR irradiation time (Fig. 6A). There are good linear relationships between signal intensities associated with disulfide bond breakage, protein hydrolysis, cytochrome c and phenylalanine release, and DNA fragmentation with irradiation time. In a previous study, Kang *et al.* also had reported the same linearity in events of disulfide bond breakage and DNA fragmentation during apoptosis induced by H_2_O_2_.^60^ To better compare the rate of the above intensity changes, we illustrated the rate of the intensity decrease against time as radar maps (Fig. 6B). As shown, MCF-7 cells exhibited the largest rate of intensity change at 500 cm^–1^ and the smallest at 750 cm^–1^. The same trend was also observed for A549 and HeLa cells (Fig. 5B, Fig. S19, 20†). These results imply that breakage of sulfate bonds produces the largest rate, while cytochrome c has the smallest one.

### Initiator of PPTT-induced apoptosis

**Fig. 7:**
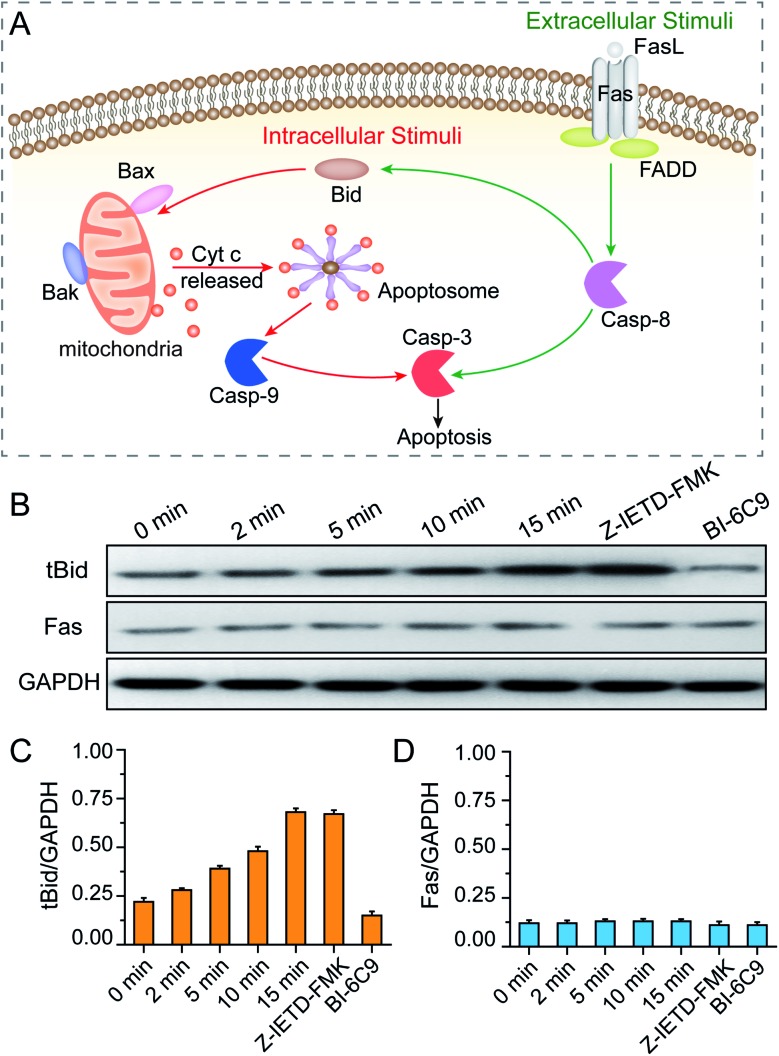
(A) Illustrations of the intrinsic and extrinsic pathways of apoptosis. (B) Western blot analysis of BID and Fas activation of MCF-7 cells during the PPTT process. GAPDH served as the loading control. The expression levels of tBID (C) and Fas (D) were quantified by densitometry and normalized to the GAPDH control. Cells were incubated with mPEG/RGD/NLS-Au NSs and irradiated under an 808 nm laser for 0–15 min. Control cells were pretreated with a BID inhibitor (BI-6C9) and a caspase-8 inhibitor (Z-IETD-FMK), respectively, and subsequently incubated with the mPEG/RGD/NLS-Au NSs, and then irradiated under an 808 nm laser for 15 min.

After revealing the molecular signaling pathway of PPTT-induced apoptosis, we then determined which molecule activates it. There are two major pathways of apoptosis, *i.e.*, extrinsic and intrinsic pathways (Fig. 7A). The extrinsic pathway is the death receptor-initiated pathway, which is activated by specific binding between a death receptor ligand (*e.g.*, FasL) and a death reporter (*e.g.*, Fas) expressed on the surface of cell membranes; these events are followed by the formation of a death-inducing signaling complex (*e.g.*, Fas/FasL) and activation of caspase 8 and caspase 3. The other pathway is the intrinsic mitochondrial pathway, which is activated by the BH3-only protein BID, which subsequently transforms into the active truncated form tBID and triggers Bak and Bax activation, mitochondrial outer membrane permeabilization and cytochrome c release. Once cytochrome c is released, it binds with apoptotic protease activating factor-1 (Apaf-1) and adenosine triphosphate (ATP), which then bind to pro-caspase 9 to create an apoptosome. The apoptosome cleaves the pro-caspase to its active form caspase-9, which subsequently activates the effector caspase-3, and finally sets off a set of events downstream that ultimately leads to apoptosis.^54^ Of note, there is crosstalk between the extrinsic and intrinsic pathways in the Fas signalling cascade; caspase-8 in the extrinsic pathway can directly activate downstream caspase or indirectly cleave BID and then translocate to the intrinsic pathway.

As elucidated from our Raman data, there is an important signature, *i.e.*, release of cytochrome c, which implies that a mitochondria-mediated pathway is involved. To verify this, mitochondria damage was monitored by measuring the loss of mitochondrial membrane potential (Δ*Ψ*_m_). As shown in Fig. S21,† more PPTT-treated cells lost their Δ*Ψ*_m_ at longer irradiation times. Finally, 89% of cells lost their Δ*Ψ*_m_ after 15 min of exposure to laser light, which is close to the percentage of apoptotic population obtained by PI and Annexin V-FITC staining (94.4%). These results confirm that PPTT-induced apoptosis undergoes an intrinsic mitochondria-mediated pathway. To determine which signaling molecule activates the mitochondrial pathway, we performed western blot analysis to determine the activation of BID and Fas. As shown in Fig. 7B and C, longer irradiation times triggered more generation of tBID, strongly suggesting that BID activation is involved in the mitochondrial pathway of PPTT-induced apoptosis. However, the Fas expression level remained constant during the irradiation, indicating that the Fas/FasL-initiated pathway is not triggered in PPTT-induced apoptosis, which is in good agreement with the results from del Pino *et al.*; they believed that the Fas/FasL pathway is engaged by extracellular FasL, which is usually expressed by cells from the immune system, and this is unlikely to be triggered during PPTT.^23^

To reconfirm this, we conducted two control experiments. First, MCF-7 cells incubated with a BID inhibitor (BI-6C9) were used as a control. The expression level of tBID in such cells shows a considerably low level compared with wild MCF-7 cells after 15 min of irradiation (Fig. 7B and C). Meanwhile, these control MCF-7 cells almost retained their viability after PPTT, as demonstrated by flow cytometry analysis (Fig. S22†). This control confirms that BID is activated during PPTT-induced apoptosis. Second, MCF-7 cells incubated with a caspase-8 inhibitor (Z-IETD-FMK) were used as another control, which showed almost the same expression level of tBID as wild MCF-7 cells after 15 min of irradiation (Fig. 7B and C), verifying that the activation of BID was not affected when caspase 8 was inhibited. Moreover, the expression level of Fas in this control remained unchanged, thereby excluding Fas activation in PPTT-induced apoptosis. Moreover, the results also imply that caspase-8 is not the upstream executor of BID activation in the mitochondria-mediated apoptosis pathway triggered by PPTT, agreeing with the previous result.^23^ Therefore, PPTT-induced apoptosis of MCF-7 cells is strongly thought to undergo an intrinsic mitochondrial pathway that is initiated by BID. It is worth noting that del Pino *et al.* previously reported the same conclusion in different cell models.^23^ Although it is inspiring, more experiments are needed to determine whether this finding holds true for other cases.

## Conclusion

In summary, we constructed the nuclear-targeting Au NSs both as photothermal conversion agents to effectively induce apoptosis of MCF-7, A549, and HeLa cells and as SERS probes to probe the SERS spectra of cells during irradiation in real-time. Through the time-dependent SERS spectra, an intrinsic mitochondria-mediated apoptosis pathway was revealed during the PPTT process. Moreover, a set of molecular events including disulfate bond breakage, protein hydrolysis, phenylalanine release, cytochrome c release, and DNA fragmentation was elucidated. Using synchronous and asynchronous 2D correlation maps combined with western blot results, the molecular signaling pathway in PPTT-induced apoptosis of MCF-7 cells was revealed to be a cascade of molecular events (including the release of cytochrome c, degradation of protein, and fragmentation of DNA) initiated by the BH3-only protein BID. We believe that this work is beneficial to better understand the cellular response to PPTT at the molecular level, as well as serving as a guide on how to perform PPTT and further drive forward its clinical application.

## Experimental

### Synthesis and surface modification of Au NSs

Typically, 3.0 g of PVP (MW = 5000) was dissolved in 15 mL of DMF. Then, 85 μL of HAuCl_4_·4H_2_O (50 mM) was added, followed by the addition of 43 μL of PVP-coated Au nanospheres with a 15 nm diameter (4.2 mM) to produce Au NSs. After stirring at room temperature for 2 h, the reaction solution was successively centrifuged, washed with ethanol and deionized (DI) water, and finally dispersed in 20 mL of DI water.

The as-prepared Au NSs were further modified stepwise with methoxypolyethylene glycol thiol (mPEG-SH, MW = 5000), cell-penetrating peptide RGD (RGDRGDRGDRGDPGC), and nuclear localization signal NLS (CGGGPKKKRKVGG) peptides, which were purchased from Sangon Bioengineering (Shanghai, China). First, 150 μL of PEG-SH (10 μM) was added to the Au NSs (0.43 nM, 10 mL), and it was stirred overnight. To remove the excess PEG-SH, this mixed solution was centrifuged (10 000 rpm, 8 min) and dispersed in 5 mL of DI water. Next, the Au NSs modified with PEG-SH (mPEG-Au NSs) were conjugated with the peptides. Then, 1.2 μL of RGD (5 mM) and 12 μL of NLS (5 mM) peptides were added to 5 mL of PEG-Au NSs (0.72 mg mL^–1^) and incubated for 24 h. Afterwards, this mixed solution was centrifuged (10 000 rpm, 8 min) and washed with DI water. Finally, the precipitate was dispersed in PBS (0.145 M NaCl, 1.9 mM NaH_2_PO_4_, and 8.1 mM K_2_HPO_4_, pH 7.4), and the product, mPEG/RGD/NLS-Au NSs (2 mg mL^–1^), was used for further experiments. UV-vis-NIR spectra and *ζ* potential values were measured to characterize the conjugation.

### *In vitro* SERS measurements of the cells

For SERS studies, cells that were seeded on a silicon slice were incubated with 100 μg mL^–1^ mPEG/RGD/NLS-Au NSs in a cell culture medium for 24 h. Before SERS measurement, the cell culture medium was replaced by PBS. The SERS spectra of the cells were collected at different irradiation times. The Raman laser was directed into a microscope and focused on the sample using a long-working distance objective lens (50×/0.75 N.A.). The collection time of each SERS spectrum was 10 s over a spectral range from 300 to 1800 cm^–1^. The SERS spectra were recorded using a Labram HR 800 microspectrometer (Jobin Yvon) with a 785 nm laser excitation source at 10 mW.

### SERS data analysis

The SERS spectra were smoothed, the baselines were corrected, and they were normalized using LabSpec 5 before data analysis. Principle component analysis (PCA) of the SERS spectra for the MCF-7 cells during PPTT was carried out using SPSS statistics software (SPSS Inc.). Two-dimensional Raman spectra were obtained using OMNIC software. Synchronous and asynchronous correlation intensities were computed from the spectra recorded as a function of the increasing methylation level. The 2D Raman correlation plots are presented as contour maps and were constructed by drawing the contour lines every 10% from the maximum intensity of the corresponding map.

### High-performance liquid chromatography (HPLC)-MS metabolomics analysis

To analyze the metabolites of the cells treated by PPTT, the cells were sampled after exposure to an NIR laser for different time durations. The cells were quenched in a –20 °C cold, acidified organic solution and scraped off from the dishes. Typically, cells were treated by a fast (∼2 s) and efficient wash with PBS and DI water to remove the culture medium. Then, 7 mL of metabolite extraction solvents (HPLC-grade methanol : acetonitrile (ACN) : 0.5 M formic acid (FA), 2 : 2 : 1 v/v/v, –20 °C) was added to quench and lyse the cells. Afterwards, the cells were scraped off from the dish, and the cell suspension was transferred to centrifuge tubes, followed by sonication for 30 s and incubation for 15 min in ice for metabolite extraction. The cell suspension was then centrifuged at 20 400 rpm at 4 °C for 15 min. Subsequently, the sample was frozen with liquid nitrogen, freeze-dried and kept at –80 °C. Before LC-MS analysis, the dried samples were redissolved in 100 μL of DI water, diluted 10/90 (v/v) with methanol, and vortexed and centrifuged at 15 000 rpm for 10 min at 4 °C.

HPLC-MS analysis was performed with an Agilent 1260 HPLC system (Agilent Technologies, Palo Alto, CA, USA) coupled to an API 4000 mass spectrometer (Applied Biosystems Sciex, Ontario, Canada), which is equipped with an electrospray ionization (ESI) source for ion production. Organic acids and amino acids were separated on a C_18_ column (1.8 μm, 100 × 4.6 mm; Agilent, USA). The solvent was 0.1% FA and ACN, 60/40 v/v at a flow rate of 0.3 mL min^–1^ at 30 °C. The ion spray voltage was set at 5.5 kV for positive ionization and the heating gas temperature was 500 °C. Nitrogen was used as the curtain gas (10 p.s.i). The multiple reaction monitoring (MRM) experiments were conducted by monitoring the precursor ion to product ion transitions for Phe *m*/*z* 166.1–119.9 with a declustering potential (DP) of 72 V and a collision energy (CE) of –19 eV, and for Tyr *m*/*z* 182.1–136.1 with a DP of 38 V and a CE of 17 eV.

### DNA fragmentation analysis

After 15 min of PPTT treatment, fragmented DNA in MCF-7 cells was isolated *via* a DNA extraction kit (Beyotime, C0008) following the manufacturer's instructions. The eluants containing DNA pellets were electrophoresed on a 1.8% agarose gel at 80 V for 1 h. The gel was examined and photographed using an ultraviolet gel documentation system.

### Western blot analysis

PPTT-treated cells were lysed in 0.3 mL of lysis buffer (50 mM Tris–HCl (pH 8.0), 150 mM NaCl, 1% TritionX-100, 100 μg mL^–1^ PMSF). The cell lysates were centrifuged at 12 000 rpm for 20 min and then collected as the supernatant. Equal amounts of proteins were separated using 10–15% SDS-PAGE gel and blotted onto a polyvinylidene difluoride membrane. The membrane was first incubated with primary antibodies (1 : 1000 v/v) overnight at 4 °C. The primary antibodies used were anti-Fas antibody (Abcam) and rabbit polyclonal BID (Cell Signaling Technology). The membrane was rinsed and then incubated for 1 h with peroxidase conjugated secondary antibodies (1 : 10 000, Abcam). Chemiluminescence detection was performed with an ECL kit (KGP1201) from KeyGen Biotech (China).

## Conflicts of interest

There are no conflicts to declare.

## Supplementary Material

Supplementary information
