# Supplementary material for: Raman observation of a molecular signaling pathway of apoptotic cells induced by photothermal therapy
Source: Chem Sci. 2019 Oct 15;10(47):10900–10. doi: 10.1039/c9sc04389f (PMC7066574; doi:10.1039/c9sc04389f)
Supplement: Supplementary file 1 [file SC-010-C9SC04389F-s001.pdf]

Electronic Supplementary Material (ESI) for Chemical Science.
This journal is © The Royal Society of Chemistry 2019

Electronic Supplementary Information:

Raman observation of molecular signaling pathway of apoptotic cells induced by photothermal therapy

Yingfang Xing,†[1] Zhewei Cai,#[1] Meijuan Xu,§ Wenzheng Ju,§ Xiaojun Luo,† Yaojuan Hu,† Xiaoyan Liu,† Tuli Kang,† Ping Wu,*† Chenxin Cai*† and Jun-Jie Zhu*‡

†Jiangsu Key Laboratory of New Power Batteries, Jiangsu Collaborative Innovation Center of Biomedical Functional Materials, National and Local Joint Engineering Research Center of Biomedical Functional Materials, College of Chemistry and Materials Science, Nanjing Normal University, Nanjing 210097, P.R. China.

‡State Key Laboratory of Analytical for Life Science, School of Chemistry & Chemical Engineering, Nanjing University, Nanjing 210093, P.R. China.

§Key Laboratory of Department of Clinical Pharmacology, Affiliated Hospital of Nanjing University of Chinese Medicine

#Department of Chemical and Biomolecular Engineering, Clarkson University, Potsdam, NY 13699, United States.

[1] X. Xing and Z. Cai contributed equally this work.

Contents:

1. Experiments and methods

2. Calculation of Q_F

3. Photothermal induction of apoptosis

4. Figures

Fig. S1 Typical TEM and SEM image of the prepared Au NSs.

Fig. S2 Theoretical prediction of *EF* for the Au NSs.

Fig. S3 Stability of the mPEG/RGD/NLS-Au NSs in culture medium.

Fig. S4 MTT assay for cytoxicity of Au NSs.

Fig. S5 UV-vis spectra of the mPEG/RGD/NLS-Au NSs in culture medium before/after incubation with the MCF-7 cells.

Fig. S6 CLSM images of the localization of the Au NSs within MCF-7 cells.

Fig. S7 Dependence of the temperature on laser power density.

Fig. S8 Fluorescent images of the live-dead stained PPTT-treated MCF-7 cells.

Fig. S9 Dependence of the death ratio on the irradiation time for the PPTT-treated cells.

Fig. S10 Confirmation of apoptotic MCF-7 cells induced by PPTT.

Fig. S11 Flow cytometric profiles of the PPTT-treated MCF-7 cells.

Fig. S12 Populations of living, early apoptotic, later apoptotic, and necrotic cells.

Fig. S13 SERS spectra of Au NSs before/after loading within MCF-7 cells.

Fig. S14 Experimental test of EF for the mPEG/RGD/NLS-Au NSs with use of Rh 6G

Fig. S15 SERS enhancement of the mPEG/RGD/NLS-Au NSs before/after heating.

Fig. S16 LC-MS analysis of Phe and Tyr extracted from PPTT-treated MCF-7 cells.

Fig. S17 Time-dependent SERS spectra of PPTT-treated A549 cells.

Fig. S18 Time-dependent SERS spectra of PPTT-treated HeLa cells.

Fig. S19 Dependence of relative Raman band intensities of A549 cells on irradiation time.

Fig. S20 Dependence of relative Raman band intensities of HeLa cells on irradiation time.

Fig. S21 Measurement of mitochondrial membrane potential during PPTT.

Fig. S22 Flow cytometric profiles of the Bid inhibitor-treated MCF-7 cells after PPTT.

Fig. S23 Photothermal conversion stability of Au NSs.

Fig. S24 Fluorescent images of live-dead stained MCF-7 cells in control group.

1. Experiments and Methods

Chemicals

Chloroauric acid ($HAuCl_4 \cdot 4H2O$), polyvinylpyrrolidone (PVP) were purchased from Sigma-Aldrich (Shanghai, China). N, N-dimethyformamide (DMF) was purchased from Sinopharm (Beijing, China). Methoxypolyethylene glycol thiol (mPEG-SH, MW = 5000) was purchased from Yuanye Biotechnology Co. Ltd (Shanghai, China). Cell-penetrating peptide RGD (RGDRGDRGDRGDPGC), nuclear localization signal NLS (CGGGPKKKRKVGG) peptides, trypan blue staining kit, MTT cell proliferation, cytotoxicity assay kit and DNA marker (100~2000 bp) were purchased from Sangon Bioengineering (Shanghai, China). Live & dead viability/cytotoxicity assay kit for animal cells and Annexin V-FITC apoptosis detection kit was purchased from Keygen Biotech Co. Ltd (Nanjing, China). Mitochondrial membrane potential assay kit with JC-1 and apoptosis, DNA ladder extraction kit were purchased from Beyotime (Shanghai, China). Z-IETD-FMK was purchased from MedChemExpress (China). Rabbit polyclonal BID was purchased from Cell Signaling Technology (China). Antibodies against Fas, cytochrome *c* were purchased from Abcam (China).

FDTD simulation

FDTD simulations from Lumerical Solutions, Inc. (Vancouver, Canada) were performed to simulate the electric field intensity and distribution of Au NSs. We constructed a model composed of a central core and five protruding arms with tips. The geometrical structure parameters, such as core size and the number, length, and width of spikes of the model were set up according to the morphology of the as-synthesized Au NSs in TEM images. The boundary conditions of the simulation domain perfectly matched the layer absorbing boundaries. The calculation region was $0.35 \times 0.35 \times 0.35\ \mu m^3$, in which the grid resolution was set to 2 nm. Optical constants of the dielectric permittivity for gold as a function of the wavelength were adapted from the Johnson and Christy database included in the FDTD simulation.[1] The refractive index of the surrounding medium was set to be 1.33 for water.

NMR Spectra study

PVP-Au NSs, mPEG-Au NSs and mPEG/RGD/NLS-Au NSs were centrifuged (8000 rpm, 8 min) and then washed with DI water for three times to remove excess PVP, mPEG and peptides. Afterward, the purified samples were dried in vacuum and then dispersed in 500 μL D_2O. For free mPEG, RGD, and NLS, these three samples were dissolved into 500 μL D_2O. The spectra of all the samples were collected using a Bruker AV400 NMR spectrometer (USA). The spectra were analyzed by MestreNova software.

Cells culture

MCF-7 cells (human breast cancer cells), A549 cells (human lung cancer cells), and HeLa cells (human cervical cells) were obtained from the cell bank of the type culture collection of the Chinese Academy of Sciences (Shanghai, China) and cultured at 37 °C in Dulbecco's-modified Eagle medium (DMEM) supplemented with 10% fetal bovine serum (FBS), 2 mM L-glutamine, 100 U/mL penicillin, and 100 μg mL^{-1} streptomycin in a 5% CO_2 environment. After growing to 90% confluence, the cells were washed with PBS (pH 7.4), and the culture medium was replaced with 1 mL of PBS. The cell number was estimated to be $\sim 1 \times 10^5$ using a hemocytometer.

***In vitro* PPTT of the cells**

Typically, MCF-7, A549, and HeLa cells were incubated with 100 μg mL^{-1} mPEG/RGD/NLS-Au NSs for 24 h at 37 °C and rinsing with PBS, and then irradiated by an 808 nm laser with power density of 0.5 W cm^{-2} for 0–15 min. Then, the cells were stained using Live–Dead kits (Nanjing KeyGen Biotech. Ltd.) for evaluating their viability. The fluorescence images were captured under an AXIO microscope (AxioObserver A1, Carl Zeiss) equipped with epi–fluorescence and an AxioCam MRc imaging system using fluorescence mode.

MTT assay

MCF-7 cells (~1×10^4 cells each well) were first incubated with the mPEG/RGD/NLS-Au NSs of concentrations in range of 0–200 μg mL^{-1} at 37 °C for 24 h in 96-well plates, and then repeatedly rinsed with PBS to remove the excess the Au NSs. MTT solution (10 μL, 5 mg mL^{-1}, pH 7.4) was then added to each well and incubated for 4 h. Afterwards, dimethylsulfoxide (DMSO, 100 μL) was added to each well. Absorbance was recorded at 550 nm on a Synergy 2 microplate reader (Biotek). The viabilities of the cells incubated with the mPEG/RGD/NLS-Au NSs were obtained by comparing to cells before incubation.

Preparation of thin cellular sections for TEM observation

MCF-7 cells seeded onto a 150 mm dish (1 × 10^6 cells/well) were rinsed thrice with PBS and fixed with a 2.5% glutaraldehyde fixative for 4 h at 4 °C. The cells were subsequently stained with 1% osmium tetroxide for 30 min, rinsed thrice with Milli-Q water, and dehydrated in a series of graded ethanol solutions (50, 70, 95, and 100 vol %). Aided by centrifugation, the cells were then infiltrated with increasing concentrations of Epon/acetonitrile resin. The final resin-infiltrated samples were then polymerized at 100 °C for 30 min. The embedded cells were sectioned to a thickness of approximately 50–100 nm onto a water bath using an ultramicrotome (Leica EM UC 6) equipped with a 35° diamond knife and collected onto 200-mesh copper TEM grids. Cells sections were imaged and captured using a JEOL 2100 TEM.

ICP measurements

MCF-7 cells (~1×10^4 cells each well) were incubated with the mPEG/RGD/NLS-Au NSs (100 μg mL^{-1}) at 37 °C for 24 h, digested by trypsin (0.25%) containing EDTA (0.02%), centrifuged for 8 min at 1500 rpm, and washed thrice with PBS. Subsequently, the cells were treated with concentrated nitric acid to dissolve Au. The resulting solutions were left standing still at room temperature for 6 h and then heated at 60 ºC for 12 h to remove nitric acid. The residuals were dissolved in deionized water and determined by ICP using an Optima 7300 DV (Perkin Elmer).

Flow cytometric analysis

Flow cytometry analysis was performed on a FACS Calibur flow cytometer (BD Biosciences). MCF-7 cells (~1×10^6) were incubated with the mPEG/RGD/NLS-Au NSs (1 mL, 100 μg mL^{-1}) at 37 °C for 24 h, irradiated under an 808 nm laser at 0.5 W cm^{-2} for 0–15 min, washed three times with PBS, dyed with Anti-annexin V-FITC and propidium iodide (PI) for 15 min in the dark, and then detected using flow cytometry under excitation at 488 nm. The fluorescence signals of FITC and PI were recorded at emission wavelengths of 515 nm and 560 nm, respectively. Carefully adjust the logarithmic amplification scale and compensation between green and orange channels. Distinguish between viable cells (Annexin V^- / PI^-), early apoptotic cells (Annexin V^+ / PI^-), late apoptotic (Annexin V^+ / PI^+) and late necrotic cells (Annexin V^- / PI^+). In flow cytometric profiles the cells appearing in the lower left quadrant represent living cells, while the ones appearing in the lower right and upper right represent the cells in early and later apoptosis stages, respectively, and the ones in the upper left denote necrotic cells.

Measurement of mitochondrial membrane potential

Mitochondrial transmembrane potential (ΔΨm) was measured with the mitochondrial membrane potential assay kit with JC-1 (Beyotime, Shanghai, China). PPTT-treated cells were incubated at 37 °C for 20 min with 0.5 mL of JC-1 working solution. Afterwards, the staining solution was removed by centrifugation at 600 rpm for 3–4 min, and the cells were washed twice with JC-1 staining 1 × buffer. Finally, the cells were resuspended in 0.6 mL of buffer. At least 10,000 cells were analyzed per sample on a FACS Calibur flow cytometer (BD Biosciences).

Trypan blue staining

MCF-7 cells were incubated with the mPEG/RGD/NLS-Au NSs (200 µL, 100 µg mL^{-1}) at 37 °C in 96-well plates (~1×10^4 cells each well) for 24 h. Then, one well was irradiated under an 808 nm laser at a power density of 0.5 W cm^{-2} for 15 min, and another well was irradiated using 808 nm laser at 20 W cm^{-2} for 3 min. 20 µL of 0.4% (w/v) Trypan blue solution was added to the wells and left for 5 min in room

temperature. Afterwards, the bright-filed images were pictured under optical microscope (AxioObserver A1, Carl Zeiss).

LDH assay

MCF-7 cells were incubated with the mPEG/RGD/NLS-Au NSs (200 µL, 100 µg mL^{-1}) at 37 °C in 96-well plates (~1×10^4 cells each well) for 24 h. Next, the cells were irradiated under an 808 nm laser at a power density of 0.5 W cm^{-2} for 15 min, and 20 W cm^{-2} for 3 min, respectively. The plates were then equilibrated at room temperature for 30 min. CytoTox-ONE reagent (20 µL, Sigma) was added to each well, and the plates were incubated for 10 min at room temperature. The fluorescence signal at 590 nm was recorded with an excitation source of 560 nm on a Synergy 2 microplate reader. LDH release was expressed relative to the basal LDH release from untreated cells.

Caspase-3 activity assay

MCF-7 cells were incubated with mPEG/RGD/NLS-Au NSs (200 µL, 100 µg mL^{-1}) at 37 °C in 96-well plates (~1×10^4 cells each well) for 24 h. Next, the cells were irradiated under an 808 nm laser at a power density of 0.5 W cm^{-2} for 15 min, and 20 W cm^{-2} for 3 min, respectively. Then, Caspase-Glo3/7 reagent (100 µL, Promega) was added to each well, followed by incubation at room temperature for 30 min. The caspase 3 activity was evaluated by recording the luminescence of each sample using a microplate reader.

2. Calculation of Q_F

In this work, a quality factor (Q_F) was adopted to evaluate the *EF* of the nanohole array according to the simulated results, which estimates the average local electric field over the entire range of Stokes Raman shifted frequencies:[3]

$$Q_F = \frac{1}{\beta}\int_{\lambda_{min}}^{\lambda_{max}} \left|\frac{E_{max}(\lambda_L)}{E_0(\lambda_L)}\right|^2 \left|\frac{E_{max}(\lambda_R)}{E_0(\lambda_R)}\right|^2 d\lambda_R \qquad (1)$$

where λ_R ranges from 804 nm to 914 nm corresponding to the Stokes Raman shift of 300–1800 cm^{-1} with a 785 nm excitation laser, and β is a normalization constant defined as $\beta = \lambda_{max} - \lambda_{min}$. λ_{max} is 914 nm, and λ_{min} is 804 nm, which means that the integral of the Raman scattering wavelengths under investigation was adopted in this work to quantify the cumulative contributions from inelastically scattered surface plasmons.

The analytical enhancement factor (AEF) was calculated to be ~1.1×10^6 for the mPEG/RGD/NLS-Au NSs with use of the equation (2),

$$\text{AEF} = (I_{SERS} / I_{RS}) \times (C_{RS} / C_{SERS}) \qquad (2)$$

where I_{SERS} is the intensity of a 1504 cm^{-1} vibration mode in SERS of Rh6G at the mPEG/RGD/NLS-Au NSs, I_{RS} is the Raman intensity of the same mode for Rh6G in the bulk solution (the Raman spectrum of Rh6G in solution is depicted in Fig. S14). The value of I_{SERS} and I_{RS} is 26450 and 247, respectively. C_{SERS} and C_{RS} are the concentration of Rh6G used for SERS and normal Raman signal recording, which are 0.1 μM and 1 mM, respectively.

3. Photothermal induction of apoptosis

The mPEG/RGD/NLS-Au NSs were used as photothermal agents for heating the cells under NIR irradiation. Since the temperature elevation under NIR irradiation sensitively depends on the irradiation power density, the temperature in suspension of cells loaded with mPEG/RGD/NLS-Au NSs was monitored under various irradiation power densities. As depicted in Fig. S7, a more rapid and greater temperature increase was observed for a longer irradiation time and a higher laser power intensity. Considering the low-energy irradiation strategy and threshold temperature of 45 °C for clinical applications,[2] a 0.5 W cm^{-2} irradiation density (808 nm laser) was used when performing *in vitro* PPTT. Of note, the continuous heating up and cooling down caused no changes to the photothermal properties of the mPEG/RGD/NLS-Au NSs (Fig. S23), which indicates their good photothermal stability.

The therapeutic effect on MCF-7 cells was first investigated by the live-dead staining results. As depicted in Fig. S8, PPTT induced a significant death of cells upon 15 min of irradiation. The MTT analysis revealed that the death ratio of PPTT-treated cells finally reached 98.0% (Fig. S9). However, the control cells (MCF-7 cells without incubation of Au NSs but with irradiation under the same conditions) almost remained entirely alive upon irradiation (Fig. S24). To determine if the cell death pathway was apoptosis, we performed a flow cytometry measurement. PPTT-treated cells were stained with propidium iodide (PI) and fluorescein isothiocyanate (FITC)-conjugated Annexin V (Annexin V-FITC). The fluorescence signals of FITC and PI were recorded at emission wavelengths of 515 nm and 560 nm, respectively. The late apoptotic will show Annexin V^+ / PI^+ result, appearing in upper right panel of flow cytometric profiles (Fig. S11).

Reference

1 P. B. Johnson and R. W. Christy, *Phys. Rev. B: Solid State*, 1972, **6**, 4370−4379.

2 A. Kumar, S. Kumar, W.-K. Rhim, G.-H. Kim and J.-M. Nam, *J. Am. Chem. Soc.*, 2014, **136**, 16317−16325.

4. Figures

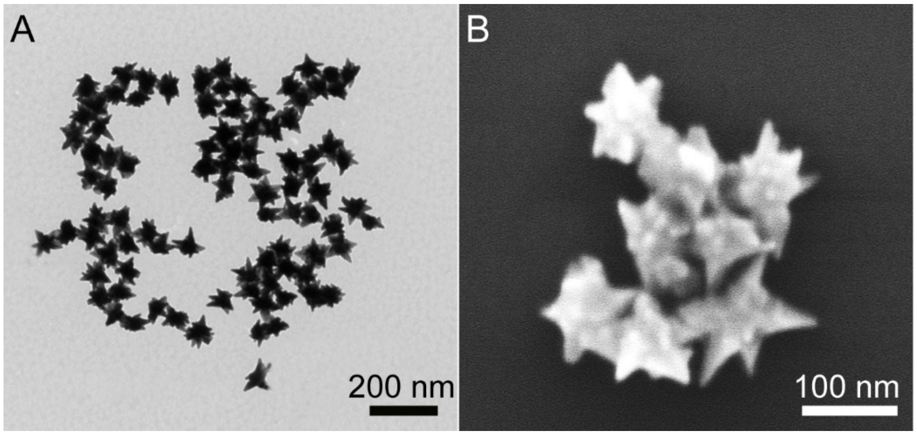

Fig. S1. Typical (A) TEM and SEM (B) image of the prepared Au NPs.

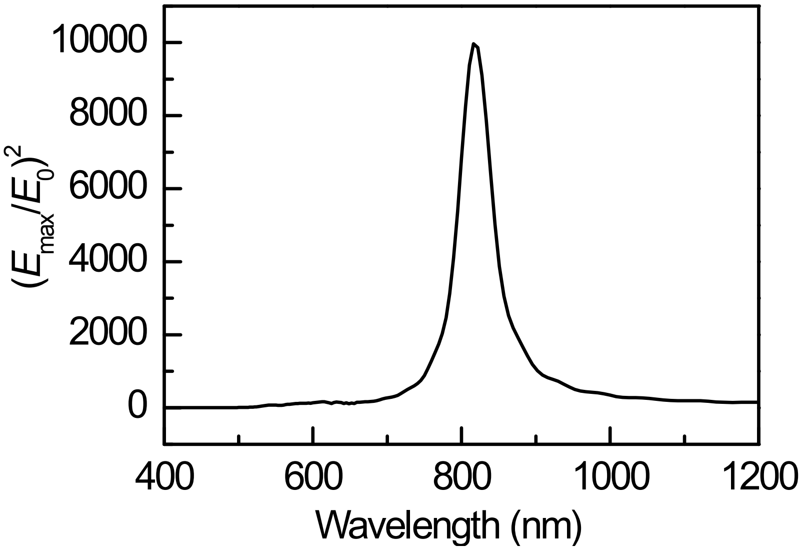

Fig. S2. Maximum electric field intensity $(E\text{max}/E_0)^2$ at the Au/H_2O interface of the Au NSs as a function of the wavelength.

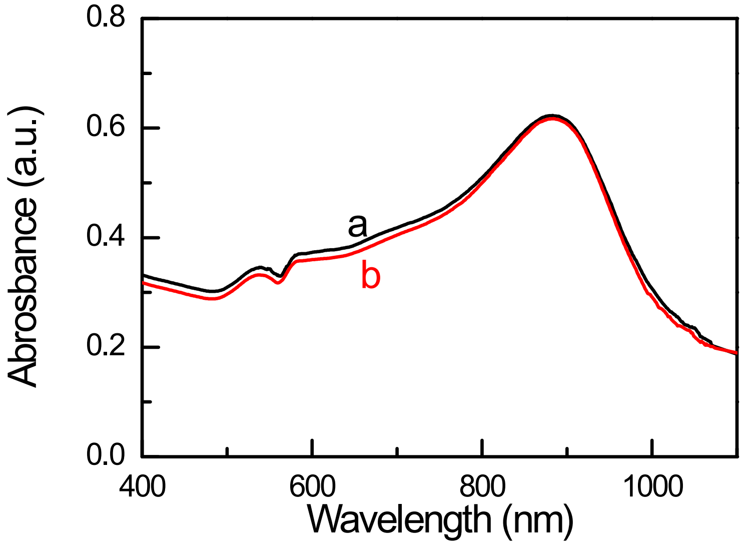

Fig. S3. UV-vis-NIR spectra of the mPEG/RGD/NLS-Au NSs suspended in culture medium (a) and stored for 7 days (b). The concentration of the mPEG/RGD/NLS-Au NSs suspension is 500 mg/mL.

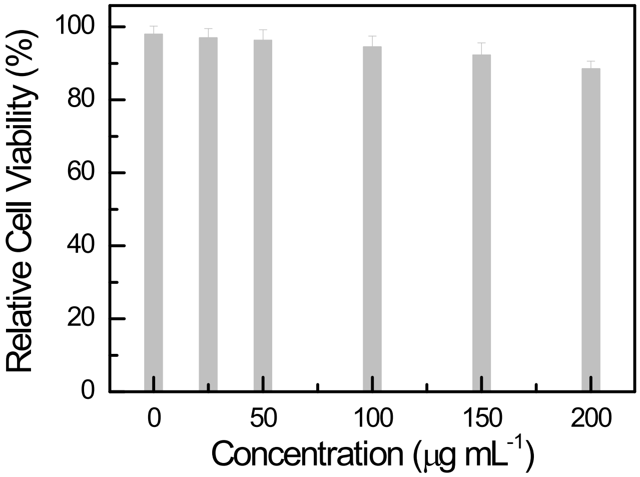

Fig. S4. Cell viability of MCF-7 cells upon incubation of mPEG/RGD/NLS-Au NSs at concentration ranging from 0 to 200 μg mL^{-1}.

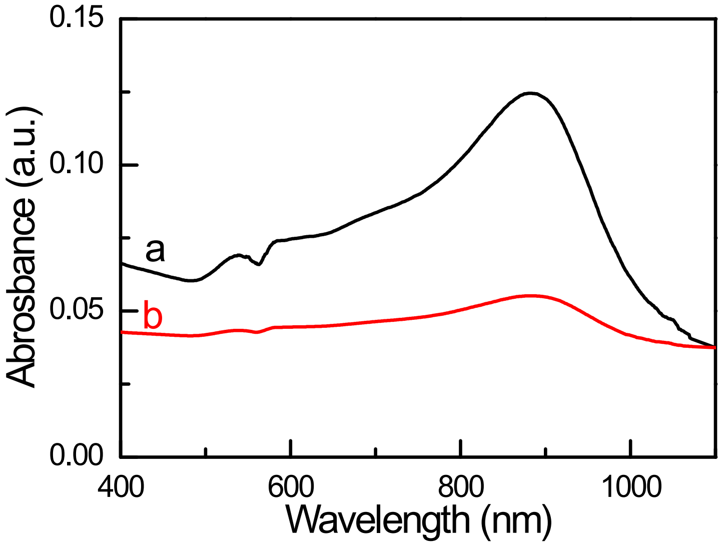

Fig. S5. UV-vis-NIR spectra of the mPEG/RGD/NLS-Au NSs (100 mg/mL) dispersed in culture media before (a) and after (b) incubation with the MCF-7 cells.

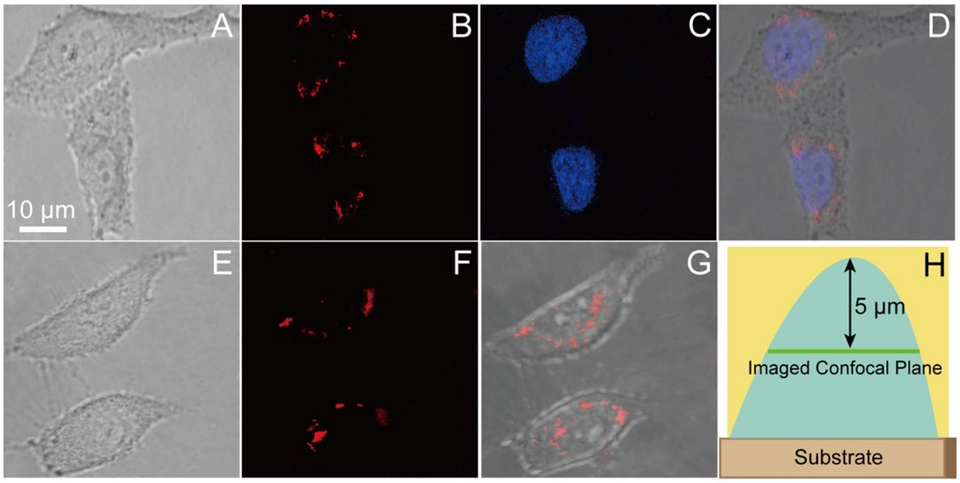

Fig. S6. (A) Bright-field image of MCF-7 cells. CLSM images of (B) MCF-7 cells loaded with Cy5/mPEG/RGD/NLS-Au NSs and (C) stained by Hoechst 33342. (D) The merged image of B and C. (E) 3D z-stack projection of MCF-7 cells loaded with Cy5/mPEG/RGD/NLS-Au NSs made of 32 images at different confocal planes with 0.5-μm decreases. (H) Schematic illustration of height of confocal planes, at the level where each CLSM image was recorded.

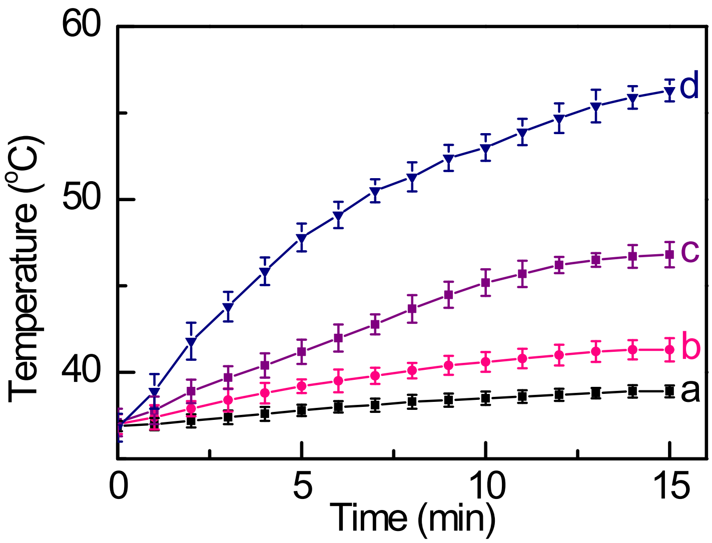

Fig. S7. Dependence of the temperature-irradiation time profiles on the power density of the mPEG/RGD/NLS-Au NSs. The laser wavelength was 808 nm, the power density was 0.1 (a), 0.25 (b), 0.5 (c), and 1.0 W cm^{-2} (d). The concentrations of the mPEG/RGD/NLS-Au NSs in solution is 100 μg mL^{-1}.

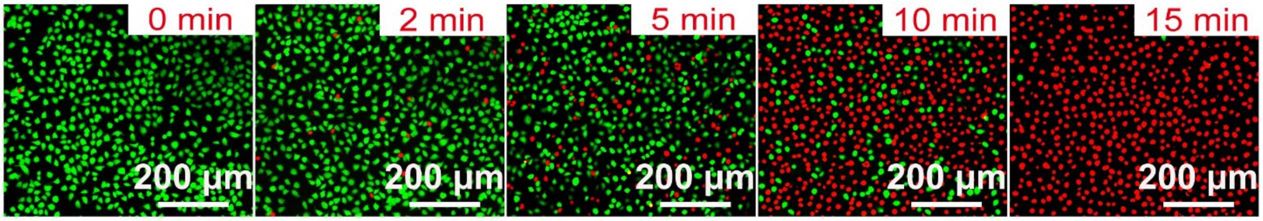

Fig. S8. Fluorescent images of live-dead stained MCF-7 cells without incubation of the mPEG/RGD/NLS-Au NSs under irradiation (808 nm, 0.5 W cm^{-2}) for 0–15 min.

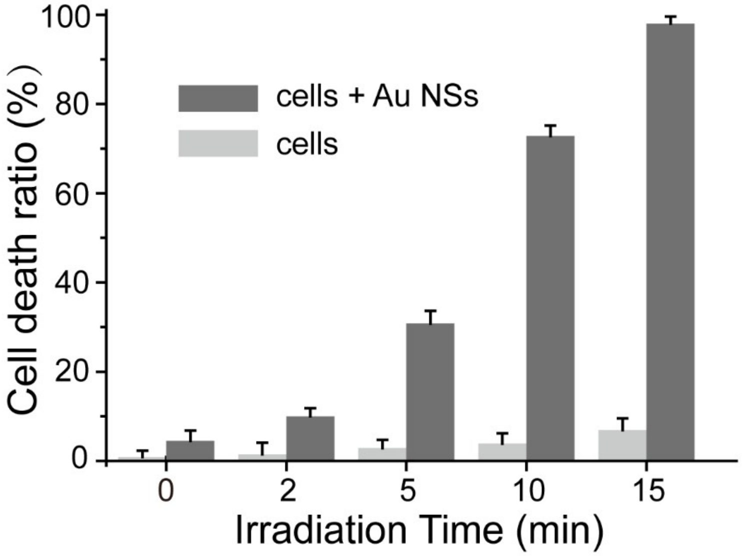

Fig. S9. Dependence of the death ratio on the irradiation time for the cells incubated with and without the mPEG/RGD/NLS-Au NSs.

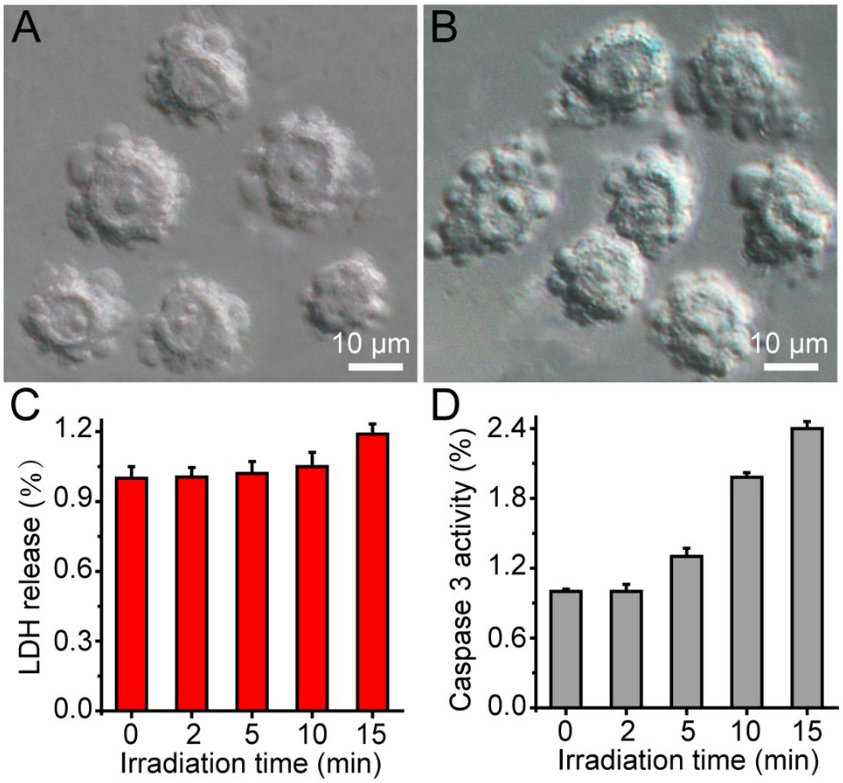

Fig. S10. (A) DIC images of apoptotic MCF-7 cells. (B) Bright-field images of apoptotic MCF-7 cells stained with Trypan blue. (C) Effects of irradiation time on LDH release level for apoptotic MCF-7 cells. (D) Effects of irradiation time on Caspase 3 activity for apoptotic MCF-7 cells.

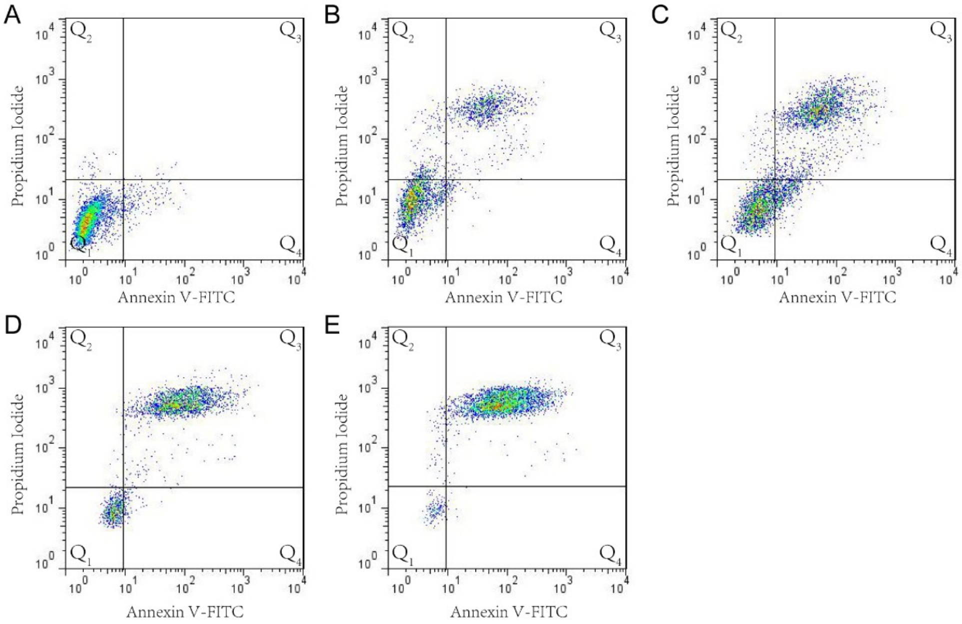

Fig. S11. Flow cytometric profiles of MCF-7 cells incubated with mPEG/RGD/NLS-Au NSs (100 μg mL^{-1}, 24 h) under irradiation (808 nm, 0.5 W cm^{-2}) for 0–15 min. The irradiation time is (A) 0, (B) 2, (C) 5, (D) 10, and (E) 15 min. The cells appearing in the lower left quadrant represent living cells, while the ones appearing in the lower right and upper right represent the cells in early and later apoptosis stages, respectively, and the ones in the upper left denote necrotic cells.

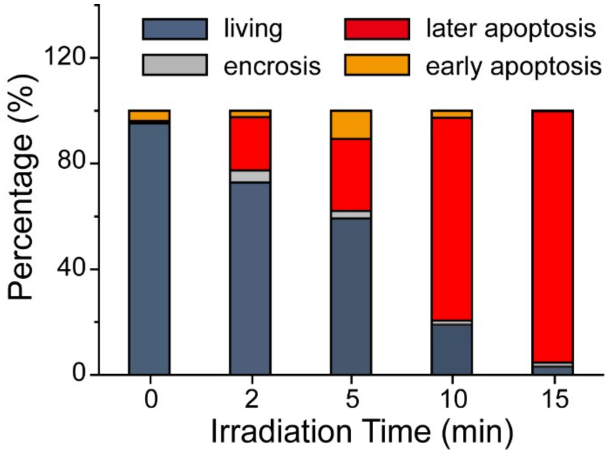

Fig. S12. Dependence of the percentage of living, early apoptotic, later apoptotic, and necrotic cells on the irradiation time. The concentration of the mPEG/RGD/NLS-Au NSs was 100 μg mL^{-1}, the laser wavelength was 808 nm, and the power density was 0.5 W cm^{-2}.

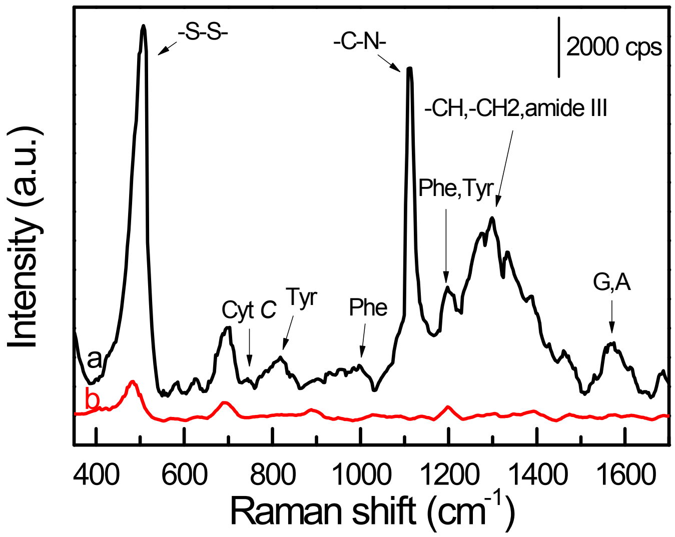

Fig. S13. SERS spectra of the mPEG/RGD/NLS-Au NSs after (curve a) and before (curve b) loading within MCF-7 cells.

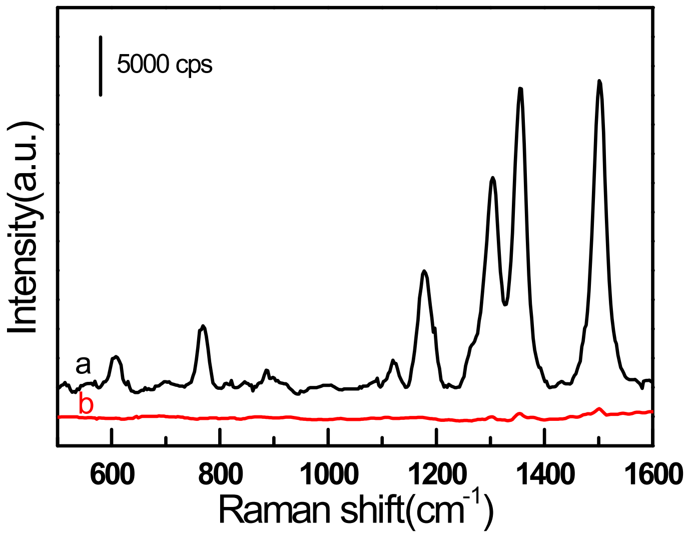

Fig. 14. SERS spectrum of Rh 6G (0.1 μM) on mPEG/RGD/NLS-Au NSs Au NSs (a); Raman spectrum of Rh 6G (1 mM) bulk solution on mica substrate (b). The excitation is 785 nm.

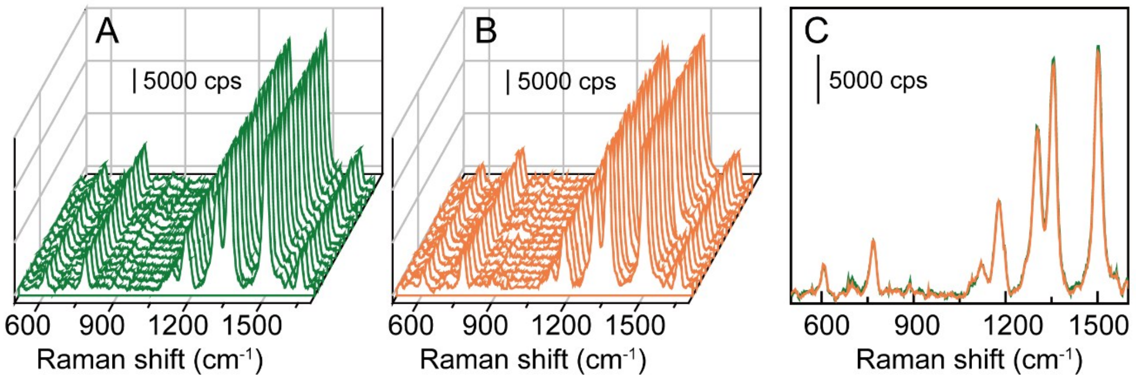

Fig. 15. SERS spectra of Rh 6G (0.1 μM) on mPEG/RGD/NLS-Au NSs before (A) and after exposure to 808 nm laser at 0.5 W cm^{-2} for 15 min (B). The excitation is 785 nm. (C) Comparison of the SERS spectra of Rh 6G on mPEG/RGD/NLS-Au NSs before/after heating.

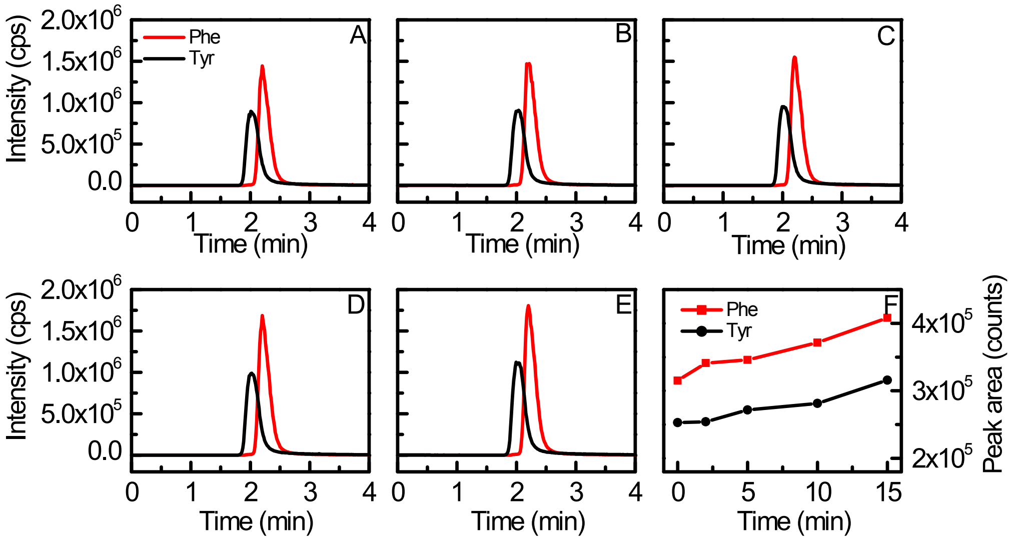

Fig. S16. LC-MS analysis of Phe and Tyr extracted from PPTT-treated MCF-7 cells. MCF-7 cells were incubated with the mPEG/RGD/NLS-Au NSs (100 μg mL^{-1}) and irradiation under an 808 nm laser at 0.5 W cm^{-2} for 0 (A), 2 (B), 5 (C), 10 (D), and 15 min (E). (F) The dependence of the amount of Phe and Try as a function of the irradiation time.

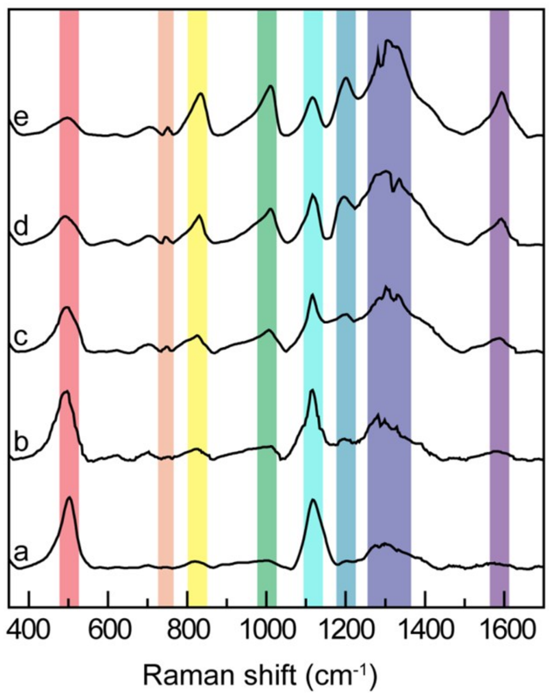

Fig. S17. Normalized SERS spectra of A549 cells incubated with mPEG/RGD/NLS-Au NSs (100 μg mL^{-1}) under an 808 nm laser (0.5 W cm^{-2}) for 0 (a), 2 (b), 5 (c), 10 (d), and 15 min (e).

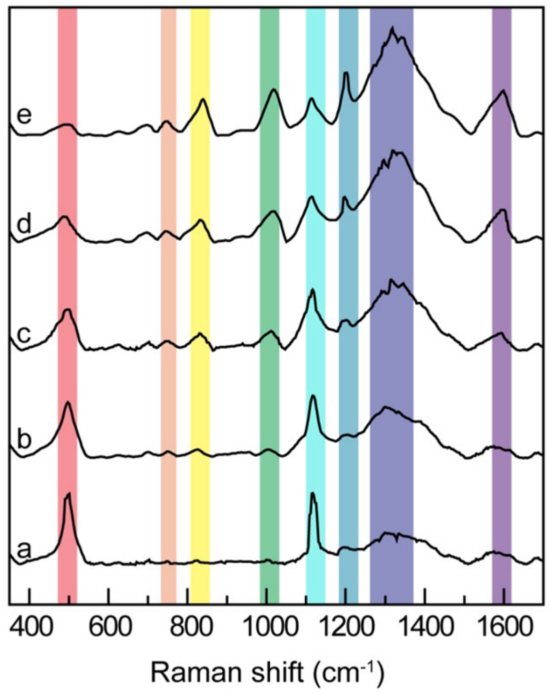

Fig. S18. Normalized SERS spectra of HeLa cells incubated with mPEG/RGD/NLS-Au NSs (100 μg mL^{-1}) under an 808 nm laser (0.5 W cm^{-2}) for 0 (a), 2 (b), 5 (c), 10 (d), and 15 min (e).

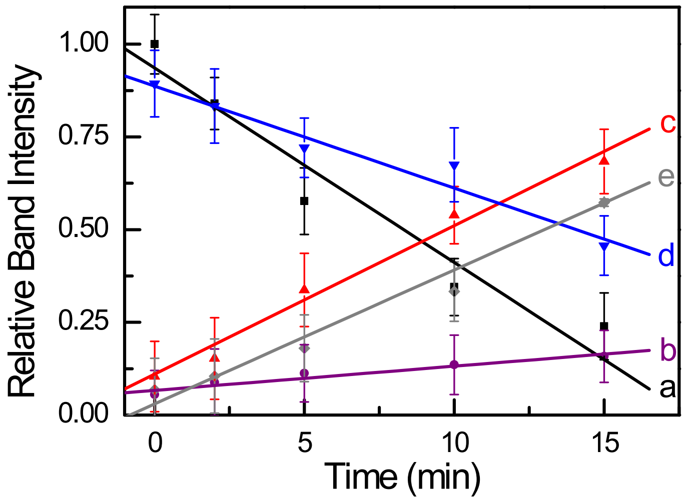

Fig. S19. Dependence of relative Raman band intensities at 500 (a), 750 (b), 1000 (c), 1120 (d), and 1586 cm^{-1} (e) in SERS spectra of A549 cells as a function of irradiation time.

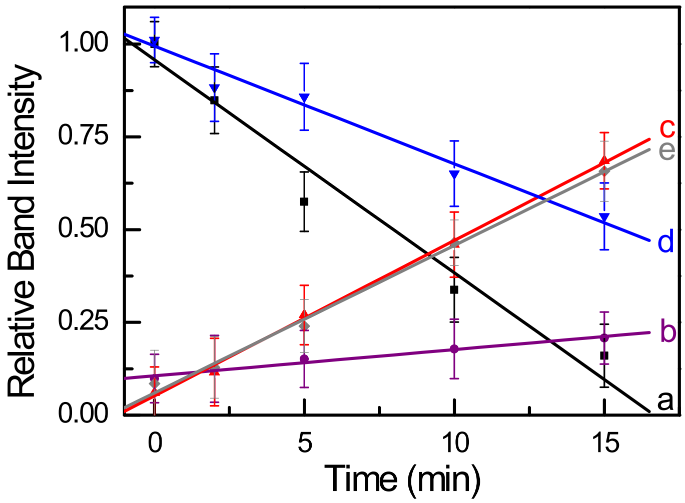

Fig. S20. Dependence of relative Raman band intensities 500 (a), 750 (b), 1000 (c), 1120 (d), and 1586 cm^{-1} (e) in SERS spectra of HeLa cells as a function of irradiation time.

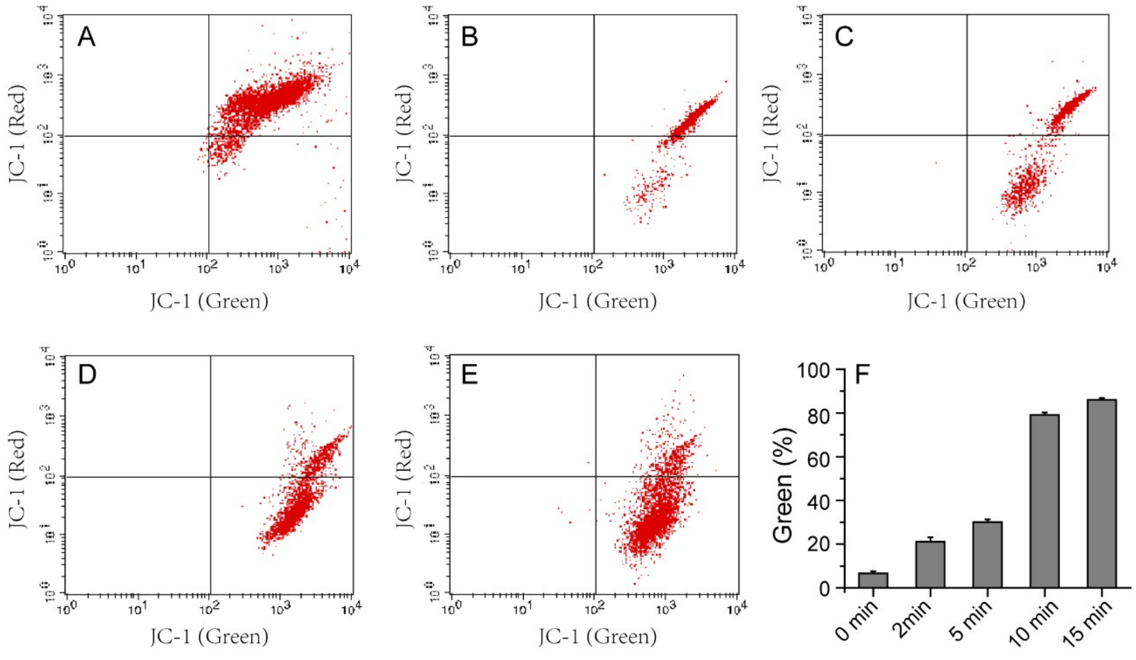

Fig. S21. The JC-1 staining results of flow cytometric profiles of MCF-7 cells incubated with the mPEG/RGD/NLS-Au NSs (100 μg mL^{-1}, 24 h) and irradiated under an 808 nm laser (0.5 W cm^{-2}) for 0–15 min. The irradiation time is (A) 0, (B) 2, (C) 5, (D) 10, and (E) 15 min. (F) Dependence of the percentage of cells with Green (%) on the irradiation time.

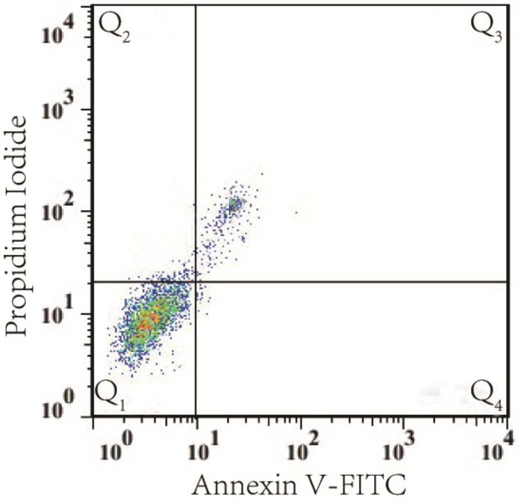

Fig. S22. Flow cytometric profiles of the Bid inhibitor-treated MCF-7 cells after PPTT. The cells appearing in the lower left quadrant represent living cells, while the ones appearing in the lower right and upper right represent the cells in early and later apoptosis stages, respectively, and the ones in the upper left denote necrotic cells.

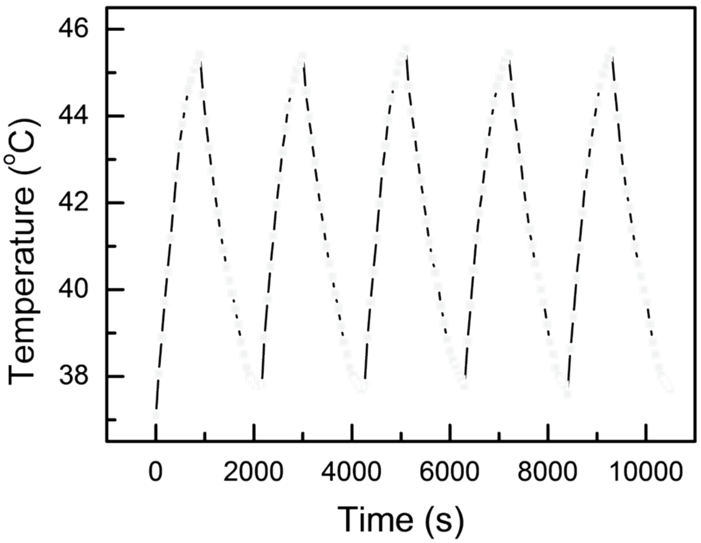

Fig. S23. Photothermal conversion stability of mPEG/RGD/NLS-Au NSs. The concentration of the mPEG/RGD/NLS-Au NSs was 100 μg mL^{-1}, the laser wavelength was 808 nm, and the power density was 0.5 W cm^{-2}.

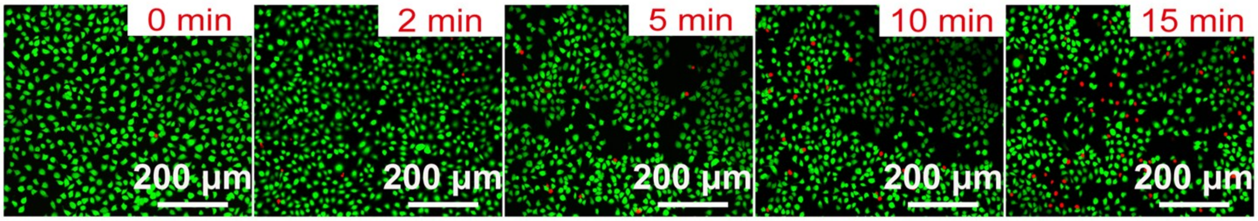

Fig. S24. Fluorescent images of live-dead stained MCF-7 cells without incubation of the mPEG/RGD/NLS-Au NSs under irradiation (808 nm, 0.5 W cm^{-2}) for 0–15 min.
